# Choroidal vasculitis as a biomarker of inflammation of the choroid. Indocyanine Green Angiography (ICGA) spearheading for diagnosis and follow-up, an imaging tutorial

**DOI:** 10.1186/s12348-024-00442-w

**Published:** 2024-12-04

**Authors:** Ioannis Papasavvas, William R. Tucker, Alessandro Mantovani, Lorenzo Fabozzi, Carl P. Herbort

**Affiliations:** 1 Centre for Ophthalmic Specialised Care (COS), Lausanne, Switzerland; 2 Moorfields Eye Hospital NHS Trust, London, UK; 3 Department of Ophthalmology, Valduce Hospital, Como, Italy

## Abstract

**Background:**

Indocyanine green angiography (ICGA) is the gold standard to diagnose, evaluate and follow up choroidal inflammation. It allows clinicians to precisely determine the type and extension of choroidal vasculitis in the two main choroidal structures, the choriocapillaris and the choroidal stroma. The presence of choroidal vasculitis is often overlooked by the physician who often does not include ICGA in the investigation of posterior uveitis.

**Purpose:**

To describe choroidal vasculitis by analysing its ICGA signs in order to investigate and follow choroiditis and determine the pathophysiological mechanisms of inflammation of choroidal vessels.

**Methods:**

The tutorial is presenting the normal findings in a non-inflamed choroid and the semiology of diverse choroidal vasculitis conditions, followed by practical illustrations using typical cases.

**Results:**

The two identified patterns of choroidal vasculitis corresponded on one side to choriocapillaritis appearing as areas of hypofluorescence depicting the involvement and extension of choriocapillaris inflammatory non-perfusion. The vasculitis of the choriocapillaris goes from limited and reversible when distal endcapillary vessels are involved such as in Multiple Evanescent White Dot Syndrome (MEWDS) to more severe involvement in Acute Posterior Multifocal Placoid Pigment Epitheliopathy (APMPPE), Multifocal Choroiditis (MFC) or Serpiginous Choroiditis (SC) with more pronounced non-perfusion causing scars if not treated diligently. On the other side, stromal choroidal vasculitis is characterised by leaking hyperfluorescent vessels that appear fuzzy and at the origin of late diffuse choroidal hyperfluorescence.

**Conclusion:**

Choroidal vasculitis is present in almost all patients with inflammatory choroidal involvement, occlusive in case of choriocapillaritis and leaky in stromal choroiditis causing vessel hyperfluorescence, fuzziness of the choroidal vessels and late diffuse stromal hyperfluorescence on ICGA. Systemic vasculitis entities produce occlusive vasculitis of large choroidal vessels.

## Introduction

Choroidal vasculitis in posterior uveitis can be divided into two categories, (1) the choriocapillary vasculitis or choriocapillaritis and (2) the stromal involvement or stromal vasculitis. The gold standard to analyse choroidal vasculitis is indocyanine green angiography (ICGA) which made it possible to subdivide choroidal vasculitis into the two patterns mentioned hereabove, each of which having characteristic signs allowing ophthalmologists to precisely determine choroidal vasculitic inflammatory involvement.

The purpose of this study was to indicate the normal appearance of the choroid on ICGA, give the basic principles on how to analyse choroidal vasculitis and characterize the signs of choroidal vasculitis or vasculopathy in non-infectious choroiditis with the help of practical clinical cases.

## Anatomy and blood flow of the choroid

**Fig. 1:**
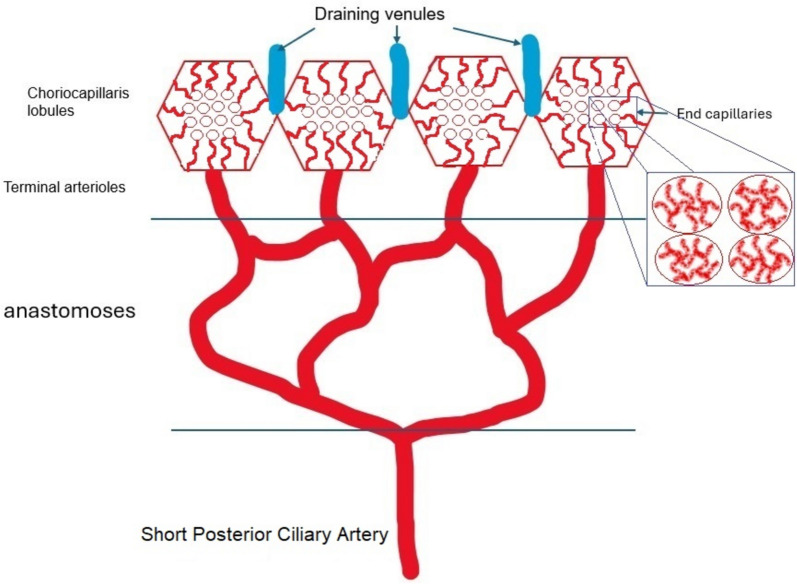
Schematic cartoon of choroidal circulation. The terminal arteriole supplies an independent lobule from the centre dividing into choriocapillaris vessels and subsequently into end-capillaries. The draining venules lie around in the periphery of the lobule. The level of inflammatory involvement of choroidal vessels determines the type of choroiditis from occlusion of posterior ciliary artery in SLE choroiditis, to staining of large stromal choroidal vessels in stromal choroiditis, to inflammatory occlusion of arterioles in serpiginous choroiditis, to inflammatory occlusions of larger choriocapillaris vessels in MFC (multifocal choroiditis) and APMPPE (acute posterior multifocal pigment epitheliopathy) and inflammatory occlusion of end-capillary choriocapillaris vessels in MEWDS (Multiple evanescent white dots syndrome)

The choroid is the most vascularized tissue in the eye and has the highest flow per gram of any other tissue in our body. The arterial supply in the choroid is segmental as is the venous drainage. Blood supply arrives via the ophthalmic artery to the branches of the anterior and posterior ciliary arteries, and further into the major choroidal arterioles via the short posterior ciliary arteries which give rise to the major choroidal arterioles. Recurrent branches of the long posterior ciliary arteries and anterior ciliary arteries contribute to the anterior choroidal blood supply. Branches of ciliary arteries lying in the outer choroidal stroma (Haller’s layer) give rise to the medium-sized choroidal arterioles of the inner choroidal stroma (Sattler’s layer). The latter arterioles supply the choriocapillaris, a dense lobular mesh with larger outer choriocapillaris vessels and smaller inner end-capillary vessels (Fig. 1). The choriocapillaris has numerous anastomotic vessels, but is functionally divided into lobules, with a central arteriolar feeder and an array of draining venules [1, 2]. The characteristic of the choriocapillaris is the fenestrated endothelium, completely permeable to both the fluorescein molecule and the large indocyanine green-protein complex. Blood drainage of the choroid starts from local groups of lobules towards venules merging with veins draining other lobules. The outer choroidal stroma contains bundles of large choroidal veins which then coalesce into vortex veins that drain into the superior and inferior orbital veins. There are usually 3–8 vortex veins per eye, the majority of individuals having 4 or 5.

## Imaging of choroidal vasculitis

### Indocyanine Green Angiography (ICGA)

ICGA is the principal imaging modality at the base of the analysis of choroidal vasculitis.

#### Principles of ICGA

ICGA relies on two essential characteristics of the indocyanine green (ICG) molecule: The first characteristic is that maximum absorption of the ICG molecule occurs at around 800 nm followed by fluorescence emission at around 830 nm which can be detected through the retinal pigment epithelium (RPE) by infrared cameras. At this point, it is important to mention that the term cyanescence is inadequate and should not be used as the basic optical mechanism which permits to see the ICG is indeed fluorescence [3].

**Fig. 2:**
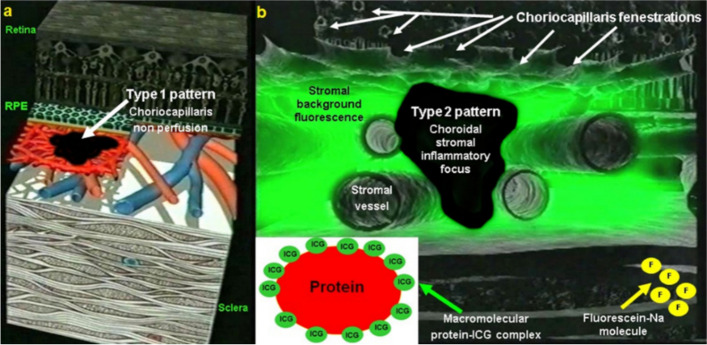
ICGA patterns. **a** Type 1 pattern of choriocapillaris non perfusion or hypoperfusion. **b** Type 2 pattern of choroidal stromal foci

The second characteristic is that the ICG molecule is bound in up to 98% to blood proteins reaching a molecular weight of 60’000 to 80’000 Daltons. This large molecular complex does not egress from inflamed retinal vessels nor from large choroidal vessels because of the tight junctions that make these vessels impermeable to large molecules. However, it physiologically egresses from the fenestrated choriocapillaris [4] and impregnates the choroidal space determining the ICGA patterns (Fig. 2). There are two ICGA patterns [5]. Firstly, inflammatory choriocapillaris non perfusion or hypoperfusion which appears as irregular geographic areas of hypofluorescence better delineated in later angiographic phases. The second ICGA pattern is characterised by round hypofluorescent dark dots (HDDs) often numerous, distributed evenly or at random generated by stromal inflammatory foci that impair the diffusion of the ICG-protein complex characterising stromal choroiditis (Fig. 2).

#### Mechanisms of choroidal vasculitis explaining ICGA signs

On one hand, choroidal vasculitis will translate into hypofluorescent signs in case of choriocapillaritis/ choriocapillary vasculitis because inflammation of these vessels will produce choriocapillaris non-perfusion of diverse extension from small dots to geographic areas of hypofluorescence [5, 6] (Fig. 2).

**Fig. 3:**
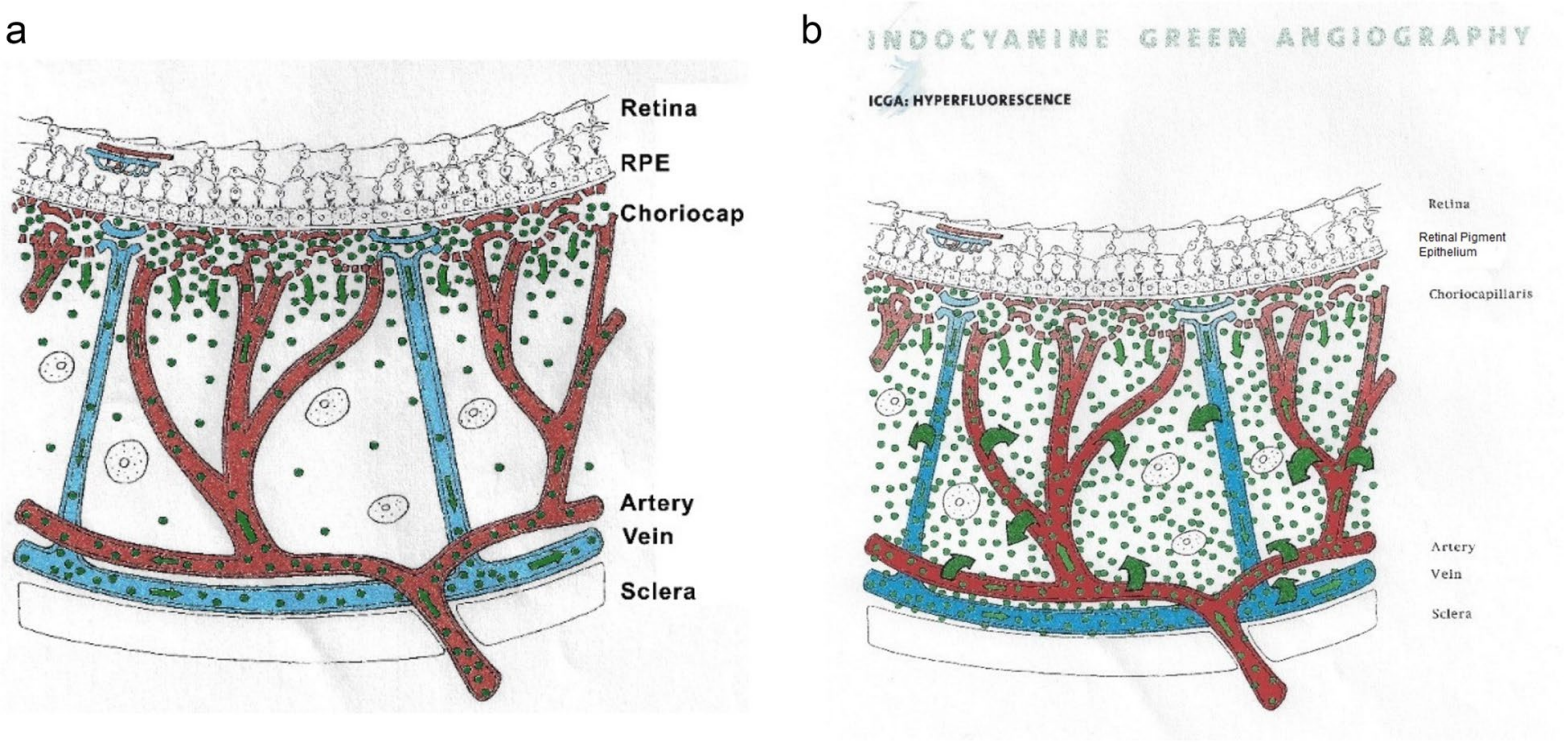
**a** Normal ICG dye circulation in the choroid. A limited number of ICG-protein complex molecules physiological egress from the fenestrated choriocapillaris and diffuse into the choroidal stroma determining faint fluorescence in the late angiographic phase. **b** Illustration of choroidal hyperfluorescence secondary to choroidal stromal vasculitis. Inflamed choroidal vessels at the origin of pathological exudation of the ICG dye causes abnormal hyperfluorescence adding up to the physiological fluorescence from ICG dye egressing normally from the fenestrated choriocapillaris

On the other hand, vasculitis related to stromal choroiditis translates into hyperfluorescent signs because exudation from larger inflamed choroidal vessels adds up to the physiological exudation of ICG from the fenestrated choriocapillaris [5, 6] (Fig. 3a).

This is very well illustrated in the cartoon of Fig. 3b showing leakage from larger vessels including arteries and veins (Fig. 3b).

Indeed, in case of stromal choroidal vasculitis the larger choroidal vessels, usually impermeable, become leaky and permeable to the ICG dye resulting in increased fluorescence. Three main signs are recognized and result from the inflammation of larger choroidal vessels, including (1) indistinct choroidal vessel (fuzziness of choroidal vessels), (2) hyperfluorescence of individual choroidal vessels and (3) diffuse late choroidal hyperfluorescence which will, in severe cases, partially or completely hide the HDDs.

#### ICGA semiology of choroidal vasculitis

Choroidal vasculitis causes two types of signs depending on which vessels are involved as explained before. In order to determine abnormal angiographic findings, it is important to show and describe normal ICGA angiographic features as shown hereunder.

##### Normal choroidal circulation on ICGA

**Fig. 4:**
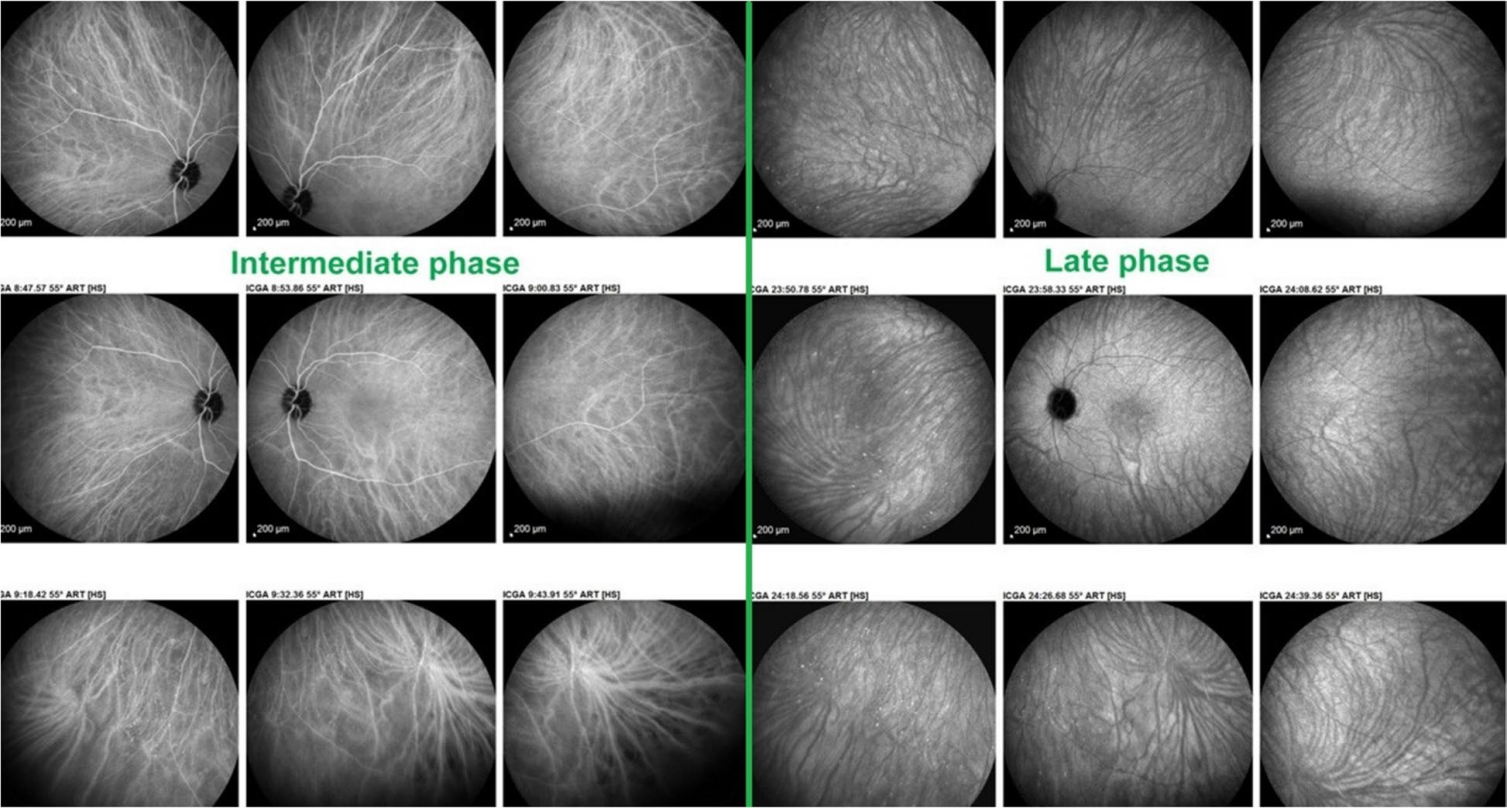
Normal choroidal vascular pattern as evidenced by ICGA. The left nine frames (left of the green separation line) show the choroidal vascular pattern in the intermediate angiographic phase (7–10’) with the ICG dye visible both in the retinal and choroidal circulations. The choroidal circulation is clearly identified with a normal aspect of vessels. The right nine frames show the choroidal vascular pattern in the late angiographic phase clearly visible in negative (no more dye intravascularly) against faint background fluorescence due to physiological dye leakage from the fenestrated choriocapillaris as the dye is no longer intravascularly

The relevant information in ICGA is principally obtained from intermediate (8–11’) and late (> 20’) angiographic phases. In the intermediate phase the ICGA dye is still within vessels (choroidal and retinal), marking both retinal and choroidal vessels appearing fluorescent. In contrast during the late phase the ICGA dye is no more within the large choroidal vessels nor in choriocapillaris vessels but outside, in the choroidal stroma, the vessels appearing in negative against the faint fluorescent background of physiologically exudated ICG into the choroidal stroma [7] (Fig. 4).

##### Dots and geographic hypofluorescent areas of inflammatory choriocapillaris non-perfusion

**Hypofluorescent dots of non-perfusion in case of Multiple Evanesent White Dot Syndrome (MEWDS)**

**Fig. 5:**
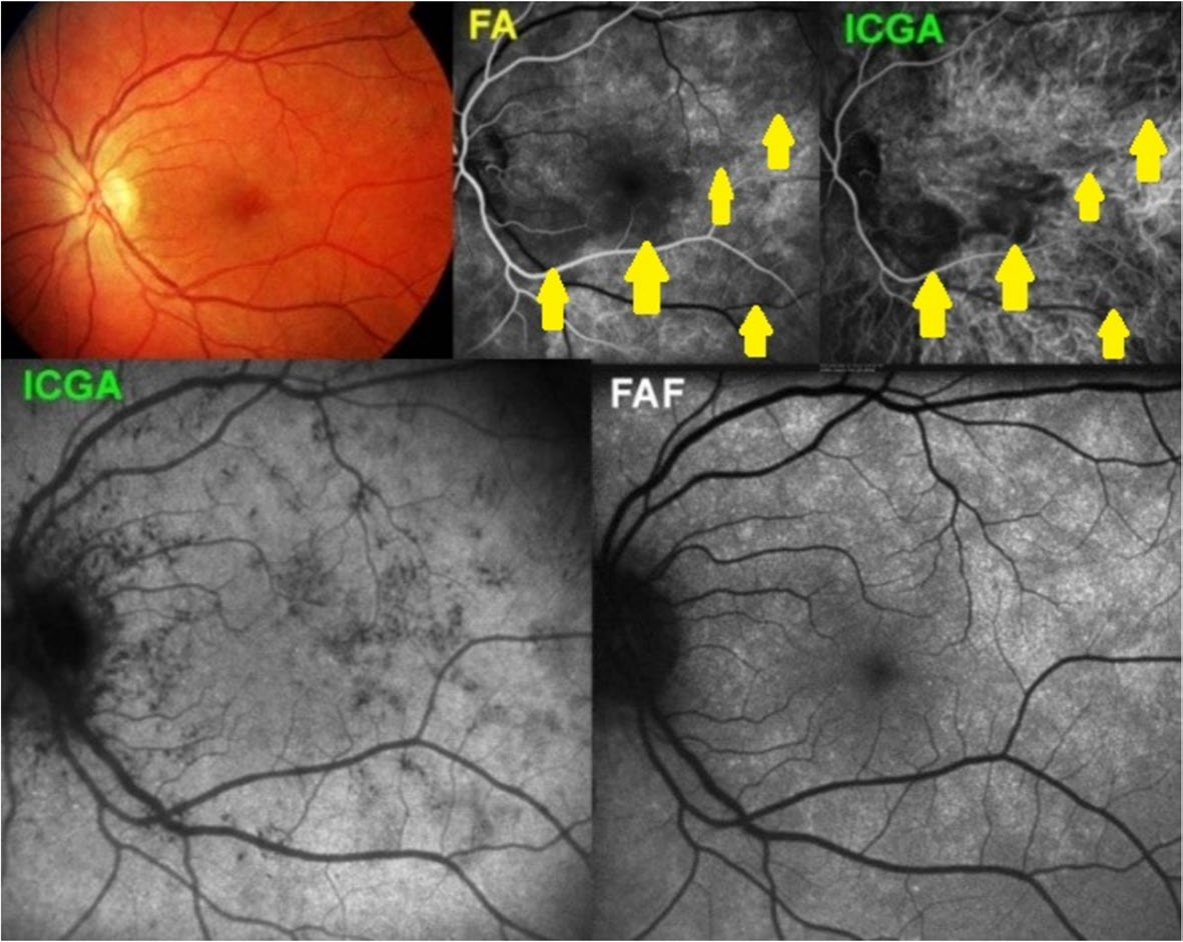
In MEWDS choriocapillary hypofluorescence is limited to dots isolated mostly non-confluent. The fundus color picture (top left) shows very faint discolorations. The early FA frame (top middle) shows choriocapillaris non-perfusion or perfusion delay (yellow arrows) which is also shown on the early ICGA frame (top right). On the late ICGA frame (bottom left) there are persistent hypofluorescent dots that correspond with certainty to choriocapillaris non-perfusion as they remain until the late angiographic phase. Bottom right, fundus hyper-autofluorescence typical of MEWDS, due to secondary loss of photopigment and/or accumulation of lipofuscin due to RPE dysfunction

The degree of extension and type of choriocapillaritis / choriocapillary vasculitis entities depend on the level and size of the vessel involved in inflammatory choriocapillaris non-perfusion. In case of involvement of small end-choriocapillary vessels such as in MEWDS, we note in ICGA the presence of hypofluorescent non-confluent dots which are more clearly visible on late angiographic phases and not the extended areas seen in APMPPE or Serpiginous [8] (Fig. 5). This is the reason why, occasionally, MEWDS has been wrongly considered as a primary photoreceptoritis. The other reason is the fact that Optical coherence tomography-angiography (OCT-A) is featureless and does not show choriocapillary drop-out, which is normal because OCT-A does not detect low-flow vessels and therefore is not sufficiently sensitive to detect end-capillary choriocapillaris circulation or the absence of it [9].

**Geographic hypofluorescent areas of choriocapillaris non-perfusion in Acute Multifocal Placoid Pigment Epitheliopathy (APMPPE)**

**Fig. 6:**
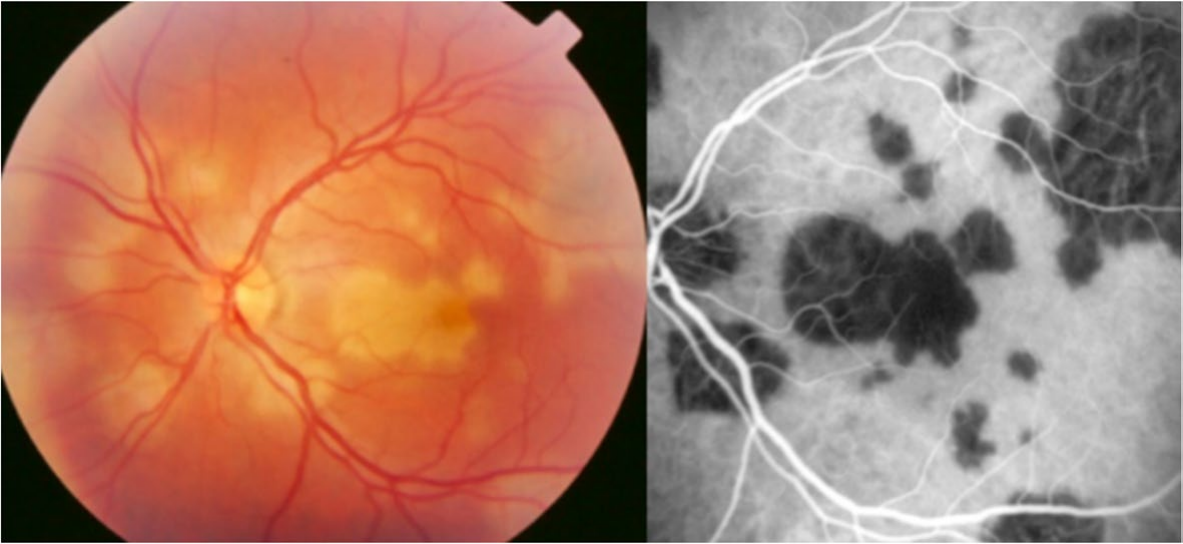
Geographic areas of choriocapillary non-perfusion in a case of APMPPE. The fundus color picture shows typical coalescent cream-coloured lesions (left picture) that correspond to extended areas of choriocapillaris non-perfusion (dark areas) on ICGA frames (right)

In contrast to MEWDS where small end-capillary vessels are inflamed, some entities show involvement of larger choriocapillaris vessels causing larger areas of inflammatory non-perfusion, which is the case of APMPPE, characterised by large and confluent areas of hypofluorescence [10] (Fig. 6).

**Geographic hypofluorescent areas of choriocapillaris non-perfusion in Serpiginous Choroiditis (SC)**

**Fig. 7:**
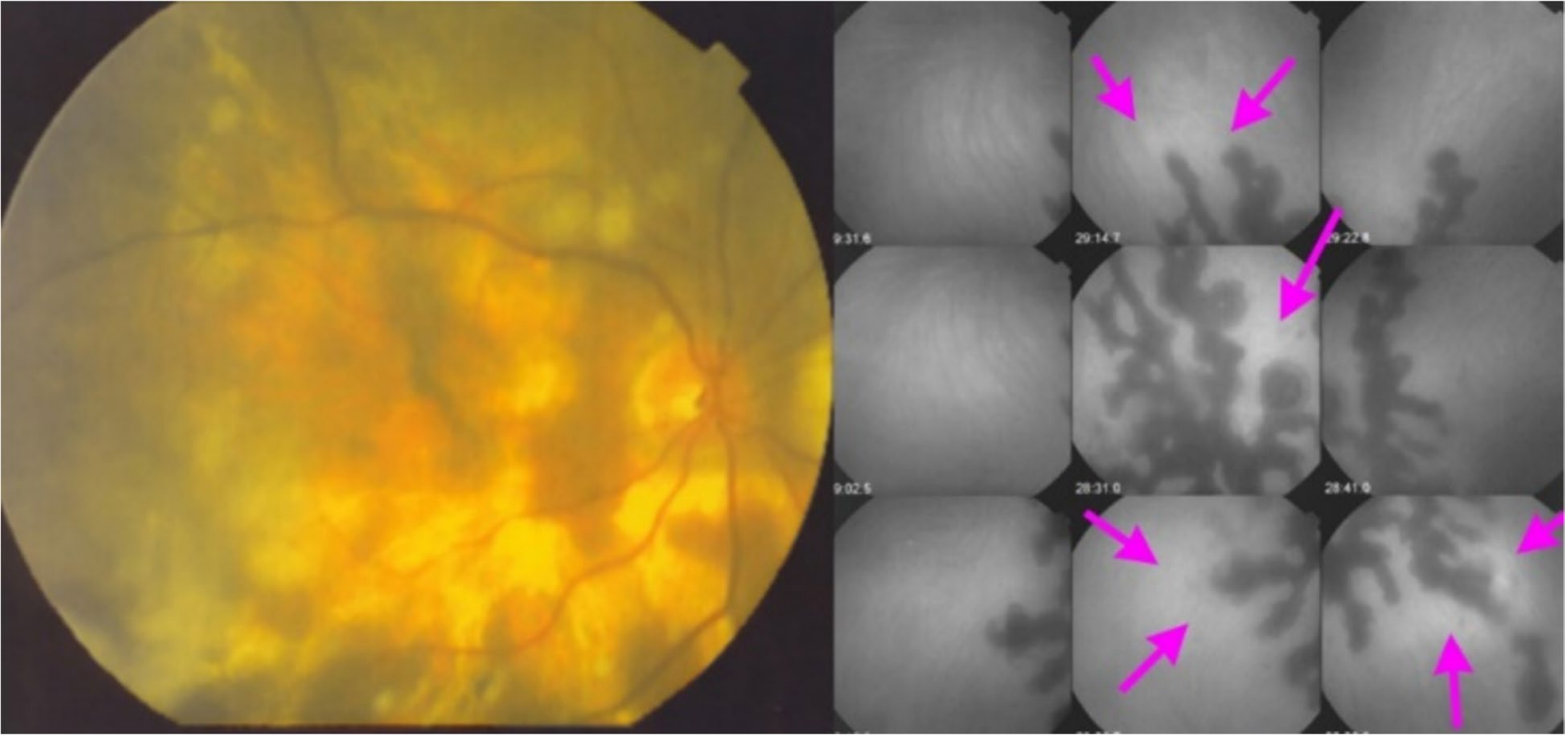
Choriocapillaris vasculitis causing serpiginous choroiditis. The fundus color picture shows snake-like areas of depigmentation (left). On the right, the ICGA frame shows the serpiginous hypofluorescent areas of non-perfusion. At the border of the progressing lesions, ICGA also shows areas of hyperfluorescence due to inflammation of vessels causing leakage (crimson arrows)

Another pattern of more extensive choriocapillaritis/choriocapillary vasculitis with creeping progression produces a serpiginous pattern of hypofluorescence [11] (Fig. 7).

##### Hyperfluorescence of stroma due to choroidal stromal vasculitis

**Early hyperfluorescence of vessels on early angiographic frames**

**Fig. 8:**
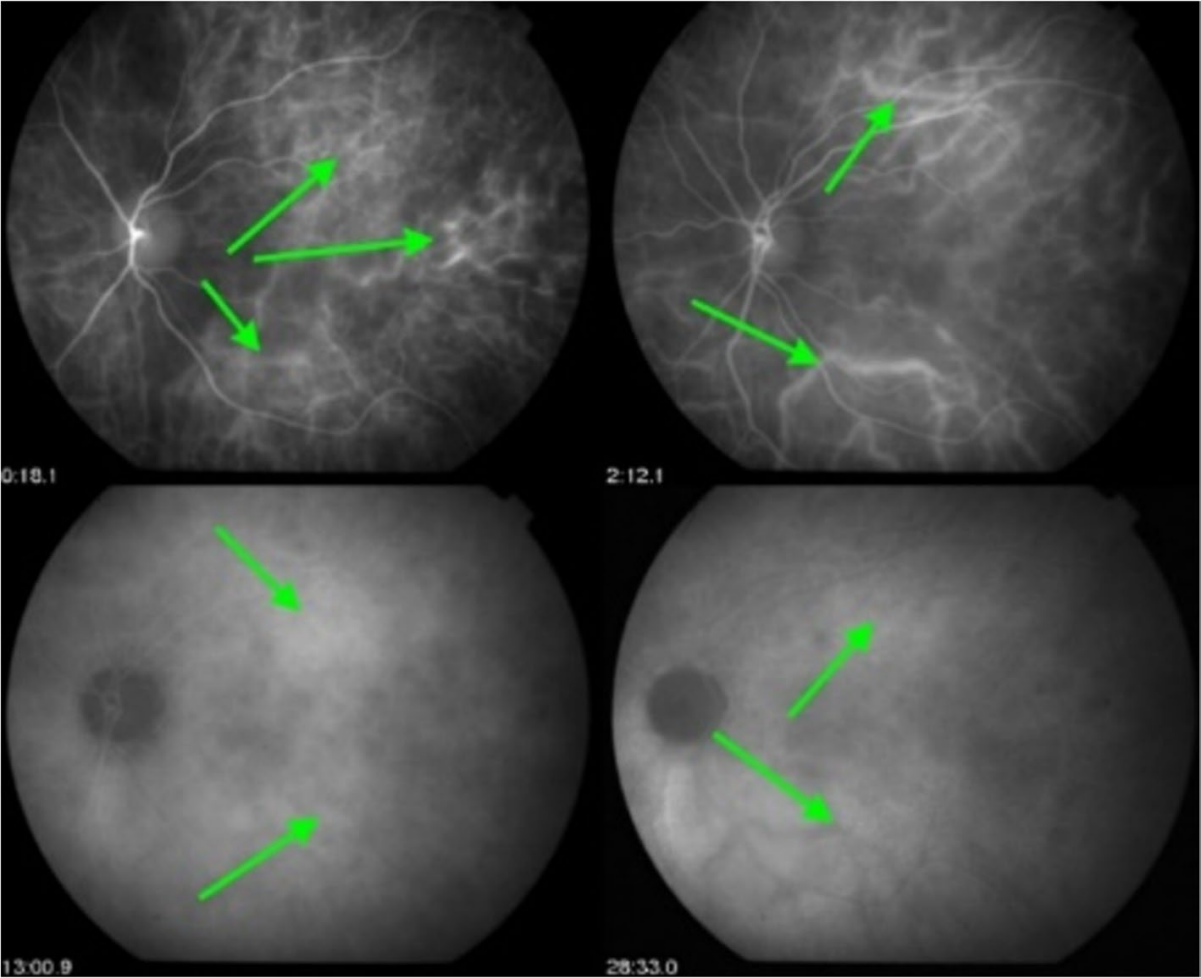
Hyperfluorescence of choroidal vessels on early ICGA frames. Early ICGA frames (top two frames) show individual inflamed/hyperfluorescent choroidal vessels (arrows) in a case of VKH disease. On intermediate and late frames (two bottom frames) there is an increased diffuse hyperfluorescence corresponding to the areas of inflamed vessels (arrows) seen on early frames

**Fig. 9:**
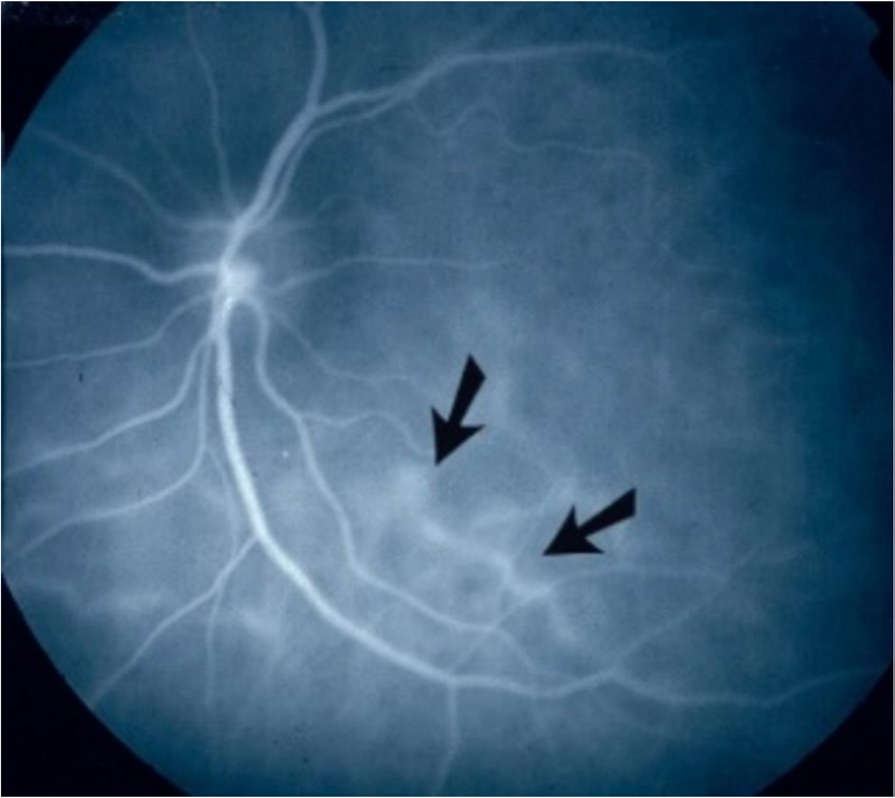
Choroidal vasculitis on early ICGA frames. Arrows show individual inflamed/hyperfluoresent vessel in a case of VKH disease

In conditions characterised by stromal choroiditis such as Vogt-Koyanagi-Harada disease (VKH) and HLA-A29 Birdshot Retinochoroiditis (BRC), ICGA can reveal, in early ICGA frames before massive dye exudation, individual hyperfluorescent vessels [12]. This sign is predominantly found in severe stromal choroiditis entities such as VKH but is rarely seen in BRC (Figs. 8 and 9).

**Fuzziness of vessels and loss of normal pattern of choroidal vessels**

**Fig. 10:**
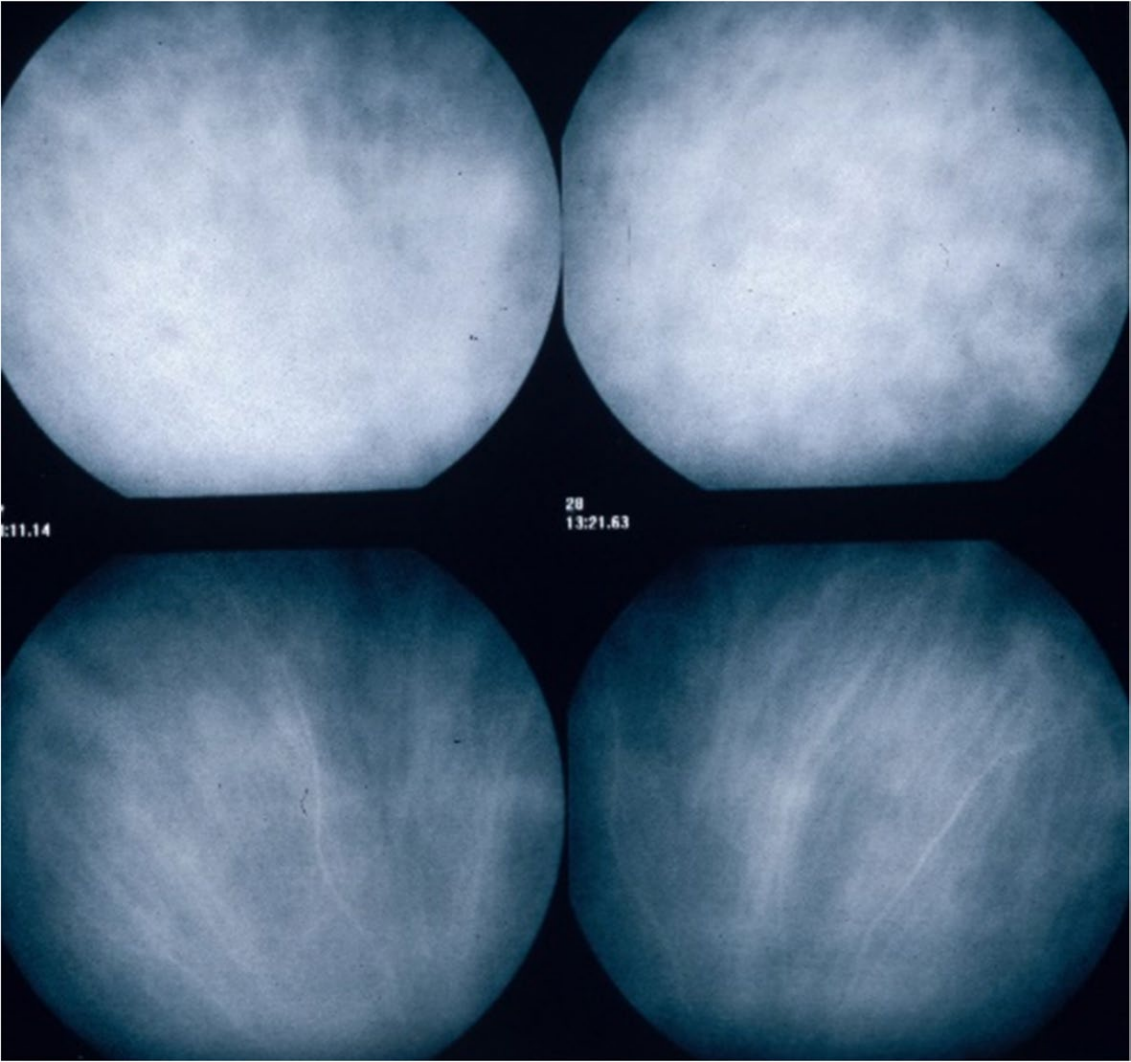
Fuzzy indistinct choroidal vessels. Normal pattern of choroidal vessels is not recognizable any longer on top frames in a case of VKH disease. After only 3 days of intravenous pulse steroids (1000 mg/day) the course of choroidal vessels is again recognizable, although still somewhat fuzzy (bottom two frames)

**Fig. 11:**
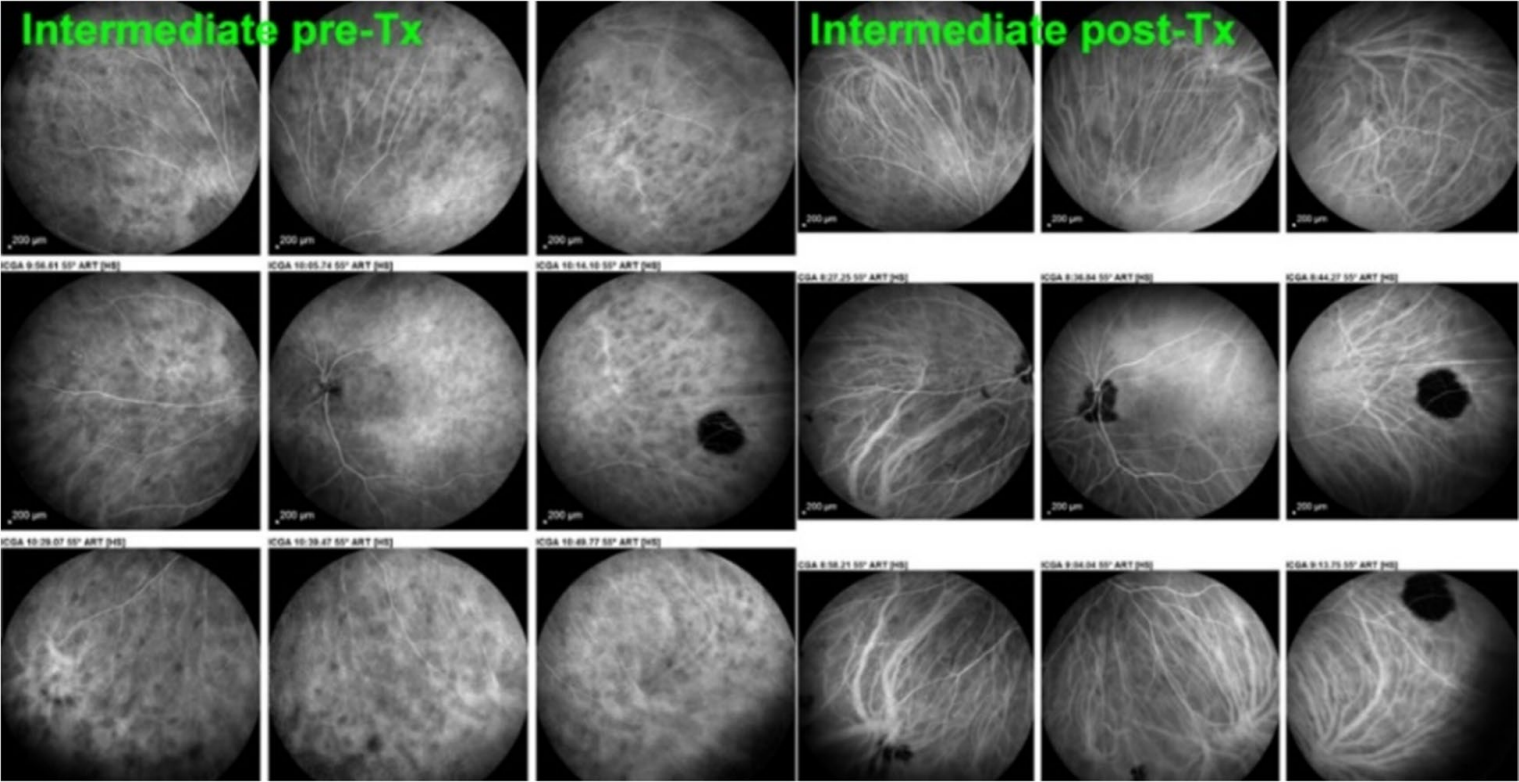
Intermediate phase inflamed vessels before and after treatment in BRC. The 9 frames on the left show numerous HDDs and loss of recognizable pattern of choroidal vessels. The 9 frames on the right were taken after treatment showing resolution of HDDs and recovery of the normal choroidal vascular pattern. The hypofluorescent round structure temporarily corresponds to a choroidal naevus

**Fig. 12:**
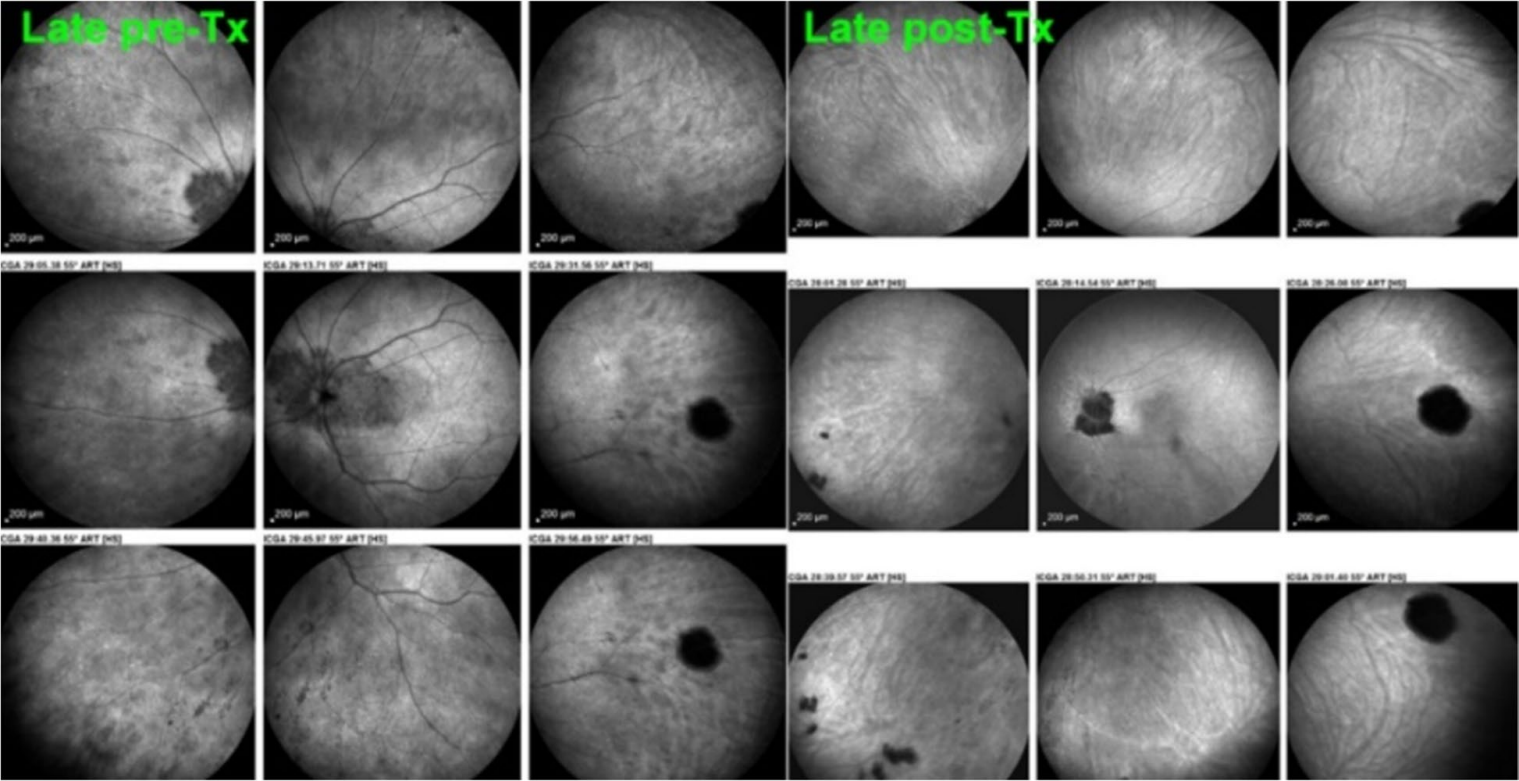
Late phase stromal inflamed vessels before and after treatment in BRC. The left 9 frames show numerous HDDs and the loss of the normal pattern of choroidal vessels that are not recognizable any longer. The 9 frames on the right show the same fundus panorama after treatment. Uninflamed vessels are now well identified in dark negative appearance and HDDs have resolved. The dark area temporally is a choroidal naevus

In stromal choroiditis (VKH, BRC), fuzzy and indistinct choroidal vessels are observed, making the normal pattern unrecognizable. After corticosteroid treatment, the course of choroidal vessels becomes recognizable again (Fig. 10). Restoration of a normal vascular pattern goes in parallel with resolution of the HDDs (Figs. 11 and 12).

**Late diffuse choroidal hyperfluorescence**

**Fig. 13:**
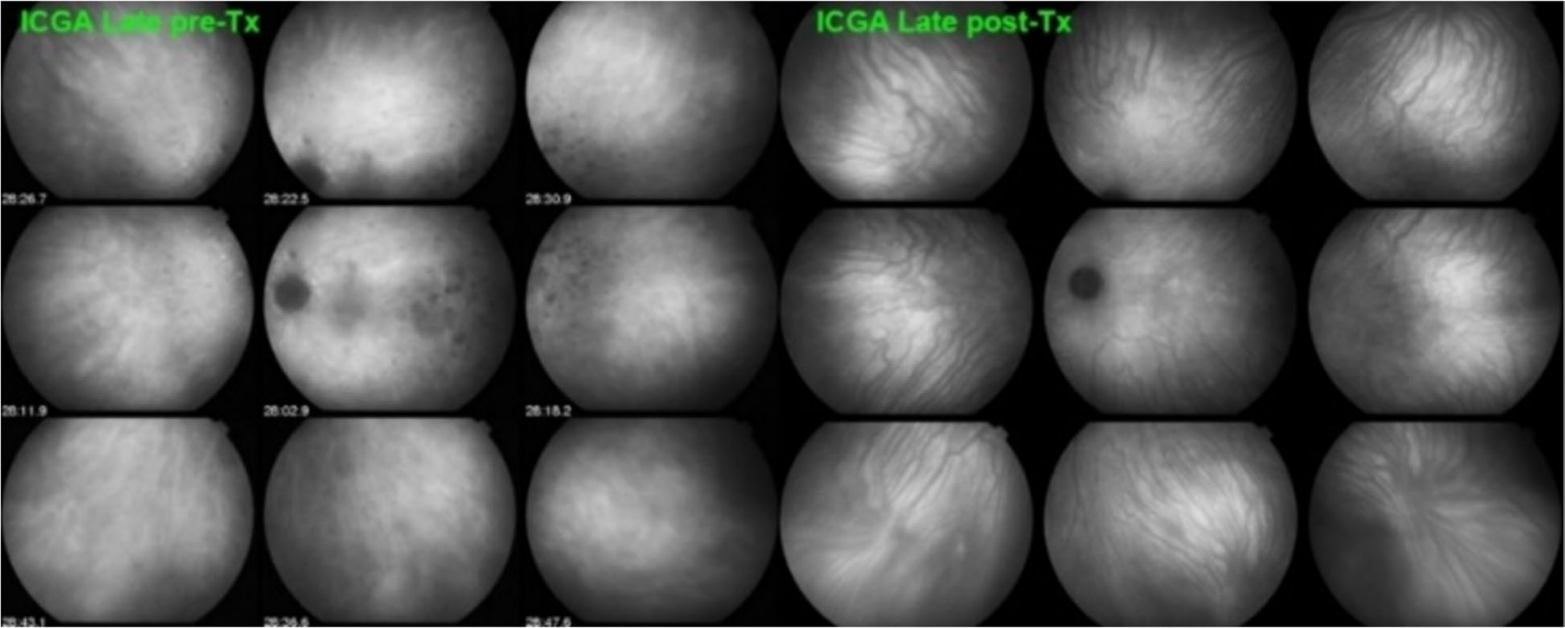
Late choroidal hyperfluorescence caused by stromal vasculitis. Case of VKH disease showing late diffuse hyperfluorescence hiding the HDDs in the initial-acute phase (left 9 frames). Note also that choroidal vessels are not recognized any longer. The 9 right frames show the same ICGA panorama after treatment with well-identified choroidal vessels appearing dark in a negative fashion

In stromal choroiditis, fuzziness of vessels in the intermediate phase produces, gives rise to diffuse choroidal hyperfluorescence in the late phase (Fig. 13).

### Multimodal imaging (other imaging modalities potentially giving complementary information on choroidal vasculitis)

#### Fluorescein Angiography (FA)

FA is used in the diagnosis and follow-up of retinal diseases and is of limited use for choroidal vasculitis. It allows a glimpse into the choroid in the first sixty seconds of angiography showing the choriocapillaris filling due to the large amount of dye present. Later, when the concentration decreases fluorescein fluorescence is blocked by the RPE. This ephemeral vision of fluorescein in the very initial phase of angiography allowed Deutman to put forward the term of choriocapillaritis for APMPPE, in the absence and before ICGA was available. He called the disease acute multifocal ischaemic choriocapillaritis (AMIC) [13].

#### Spectral Domain Optical Coherence Tomography (SD-OCT) and Enhanced Depth Imaging OCT (EDI -OCT)

OCT is a non-invasive method, easily repeatable. It is very useful in choriocapillaritis/ choriocapillary vasculitis as choriocapillaris non-perfusion and its consecutive ischaemia directly affects the outer retina, especially the photoreceptors. It is helpful to demonstrate the extent of secondary damage to the photoreceptor outer segments. In stromal choroiditis OCT is more helpful in detecting serous detachment in cases of VKH or macular oedema in cases of BRC. EDI-OCT is also helpful to detect thickening of the choroid in the early stages of stromal choroiditis and to visualise choroidal granulomas [14, 15]. 

#### Optical Coherence Tomography Angiography (OCT-A)

OCT-A is a non-invasive imaging modality characterised by a recent hype among ophthalmologists. It detects the presence or absence of flow in vessels in retina and choroid. In case of choriocapillaritis/choriocapillary vasculitis it shows capillary no-flow drop out except in end-choriocapillary vessels which cannot be imaged as significant flow is absent. In case of choriocapillaris drop-out of larger areas caused by involvement of larger vessels such as in APMPPE, MFC and SC, it is an important imaging tool for diagnosis, evaluation of the extent of lesions and follow up. For most commercially available OCT-A, the imaging field is limited to the macula, but progressively new technologies appear with extended more peripheral visualization. It is complementary to ICGA to detect the presence or absence of flow. No-flow areas can be detected in a non-invasive fashion but it is limited to the posterior pole of the fundus and is less precise than ICGA [16]. In stromal choroiditis OCT-A is not useful as the current technology cannot demonstrate the choroidal stroma. Recent studies presenting choroidal granulomas in en-face OCT-A demonstrate the top of the ‘iceberg’ of stromal foci which compress the choriocapillaris but cannot demonstrate deeper inflammatory foci as well as ICGA does.

#### Fundus Autofluorescence (FAF)

Fundus autofluorescence (FAF) is a non-invasive method, which can demonstrate RPE damage and photoreceptor pathology even at an early stage of disease. The autofluorescence signal derives from the normal lipofuscin accumulation in the RPE cytoplasm. In choriocapillaritis/choriocapillary vasculitis, patchy and/or geographic hyperautofluorescent areas is the main finding caused either by the loss of photoreceptor outer segments resulting in better visualisation of the physiological RPE autofluorescence (window defect) or damage of RPE in more severe cases producing hypoautofluorescence. The non-invasive character of the exam makes it very useful in diagnosis but more importantly for the follow-up of the inflammatory choriocapillaritis / choriocapillary vasculitis [17].

On the contrary, FAF is not useful in the early stages of stromal vasculitis where the outer retina is not yet affected.

## Pathologic entities

Table 1 lists and classifies non-infectious choroidal vasculitis.

**Table 1:** Classification of non-infectious choroidal vasculitis

1. Primary choroidal vasculitis
1.1 Primary choriocapillaritis
1.1.1. Multiple evanescent white dot syndrome (MEWDS)
1.1.2. Acute posterior multifocal placoid pigment epitheliopathy (APMPPE)
1.1.3. Idiopathic multifocal choroiditis (MFC)
1.1.4. Serpiginous choroiditis (SC)
1.2. Primary inflammation of stromal vessels (stromal choroidal vasculitis)
1.2.1. Choroidal stromal vasculitis in Vogt-Koyanagi-Harada disease (VKH)
1.2.2. Choroidal stromal vasculitis in sympathetic ophthalmia
1.2.3. HLA-A29 birdshot retinochoroiditis (BRC, choroidal involvement)
2. Secondary choroidal vasculitis
2.1. Secondary choriocapillaritis
2.1.1. Acute syphilitic posterior placoid chorioretinitis (ASPPC)
2.1.2. Tuberculosis related serpiginous choroiditis
2.2. Secondary stromal vasculitis related to ocular stromal choroiditis
2.2.1. Stromal vasculitis related to ocular sarcoidosis or tuberculosis
2.3. Choroidal (stromal) vasculitis secondary to systemic vasculitides (non-exhaustive)
2.3.1. Systemic lupus erythematosus (SLE)
2.3.2. Giant cell arteriti
3. Choroidal stromal vasculitis related to scleritis

### Primary choroidal vasculitis

#### Primary choriocapillary vasculitis (choriocapillaritis)

**Fig. 14:**
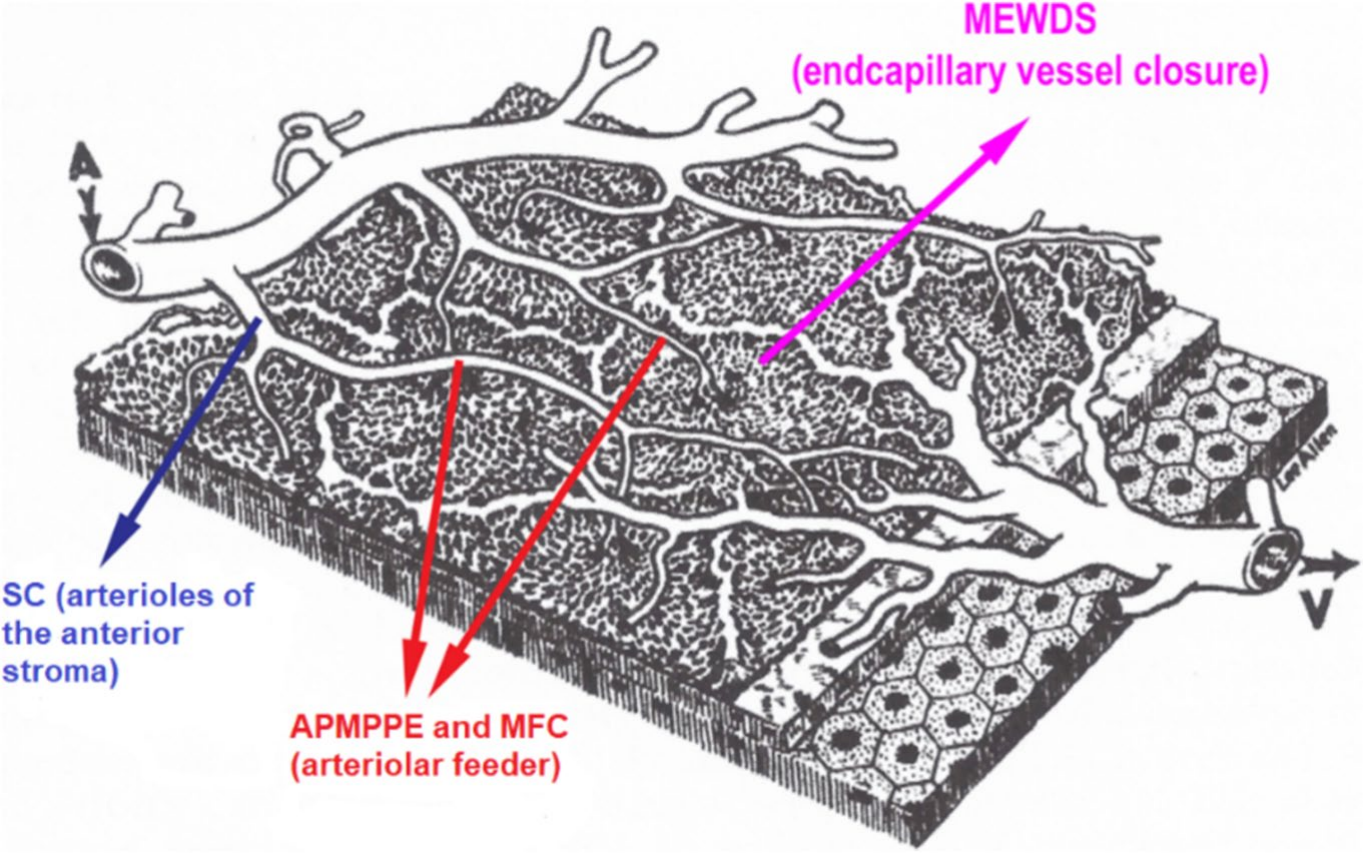
Cartoon showing choriocapillaris architecture. Depending on the size of the inflamed vessels, choriocapillaris inflammatory non-perfusion ranges from small hypofluorescent dots when endcapillary vessels are involved as in MEWDS to large hypofluorescent areas in case of MFC, APMPPE or SC

**Fig. 15:**
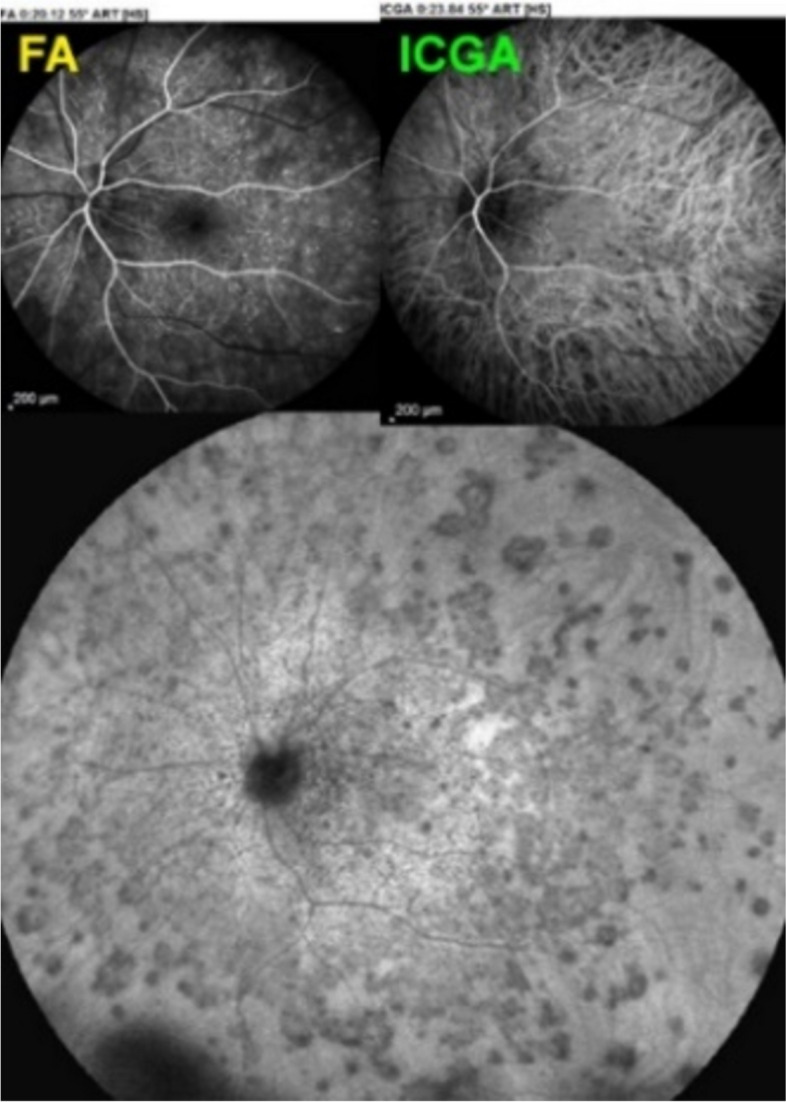
MEWDS. Young lady presenting faint visual disturbance. Typical hypofluorescent dots of non-perfusion scattered all over the posterior pole and mid-periphery in the late ICG angiographic phase. Interestingly there is an additional circulatory disturbance in delayed choriocapillaris perfusion on early FA frames and less so on ICGA beyond the temporal arcades, (2 top frames)

**Fig. 16:**
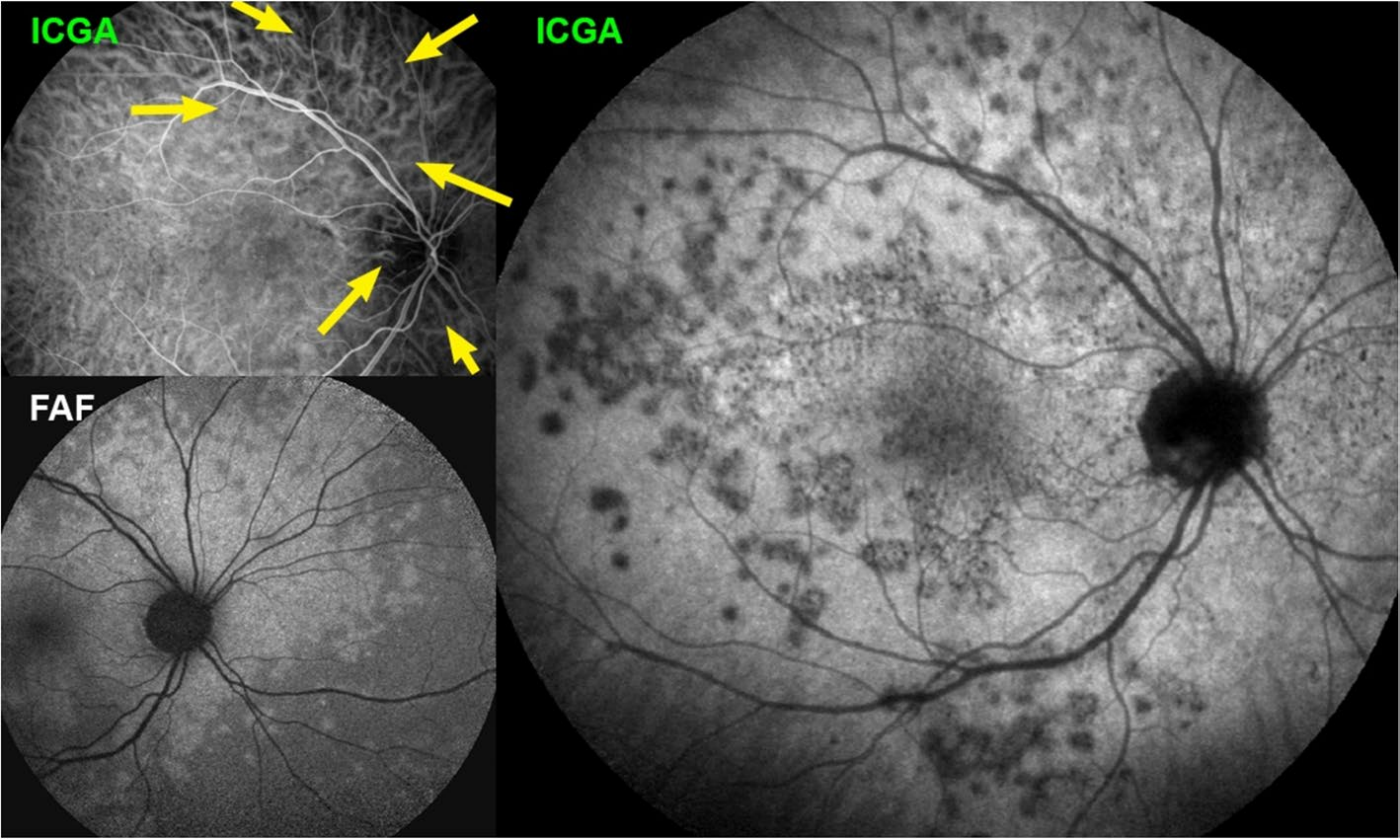
MEWDS. Young man presenting a faint subjective scotoma, Right frame: scattered areas of small dark dots representing choriocapillaris non-perfusion co-localized with FAF hyperautofluorescence caused by secondary photopigment loss (bottom left) and associated with choriocapillaris perfusion delay (top left, yellow arrows)

As mentioned before the choriocapillaris is a lobular mesh with a central arteriolar feeder. This anatomic characteristic explains the pattern of choriocapillaritis/choriocapillary vasculitis seen on ICGA. Indeed, choriocapillaritis/ choriocapillary vasculitis appears as scattered or geographic areas of hypofluorescence, indicating the lobe that is not perfused. Depending on the localization of the inflammation, in the arterial tree, there is a diverse severity of extension and involvement (Fig. 14). Very often there is a generalised choriocapillaris perfusion delay on the early frames in the area where choriocapillaris non-perfusion is seen on the late frames (Figs. 15 and 16).

##### Choriocapillary vasculitis/choriocapillaritis at the origin of Multiple evanescent white dot syndrome (MEWDS)

MEWDS is considered as the mildest form of primary choriocapillaritis / choriocapillary vasculitis, affecting the end-capillary vessels of the choriocapillaris. The disease is usually unilateral. The cause is clearly localised end-capillary hypo-perfusion and/or non-perfusion of the choriocapillaris and not primary photoreceptor disease [9] (Fig. 5). Secondary alterations of the outer retina are detected by SD-OCT corresponding to the dark hypofluorescent dots on ICGA precisely identifying the small scattered non-perfusion lesions. Corresponding hyperautofluorescent areas are attesting the secondary loss of photopigment of the outer retina. The vasculitic process in MEWDS is analysed in a few illustrative cases hereafter.

**Illustrative case of the type of choriocapillaris vasculitis present in MEWDS**

This 33-year-old lady consulted for visual disturbance of her left eye and reported to see shadows and glistening of surfaces, retaining, however, full visual acuity and discreet visual field impairment. Early FA and ICGA frames showed disturbed choriocapillaris perfusion with delay in some areas. The late ICGA frame showed numerous small non-perfusion area. It seems therefore, taking into account of the early perfusion anomalies seen in this case, (Fig. 15) that there is a more widespread perfusion problem in MEWDS apart from the typical small hypofluorescent non-perfused dots.

**Circulatory disturbances in a case of MEWDS**

This 27-year-old man complained of a subjective scotoma in his right eye with minimal visual field impairment and typical ICGA vasculitis findings of small areas of choriocapillaris non-perfusion on the late frames associated with more general choriocapillaris perfusion delay during the early frames (Fig. 16).

**Case of MEWDS showing asymmetry between FAF and ICGA**

**Fig. 17:**
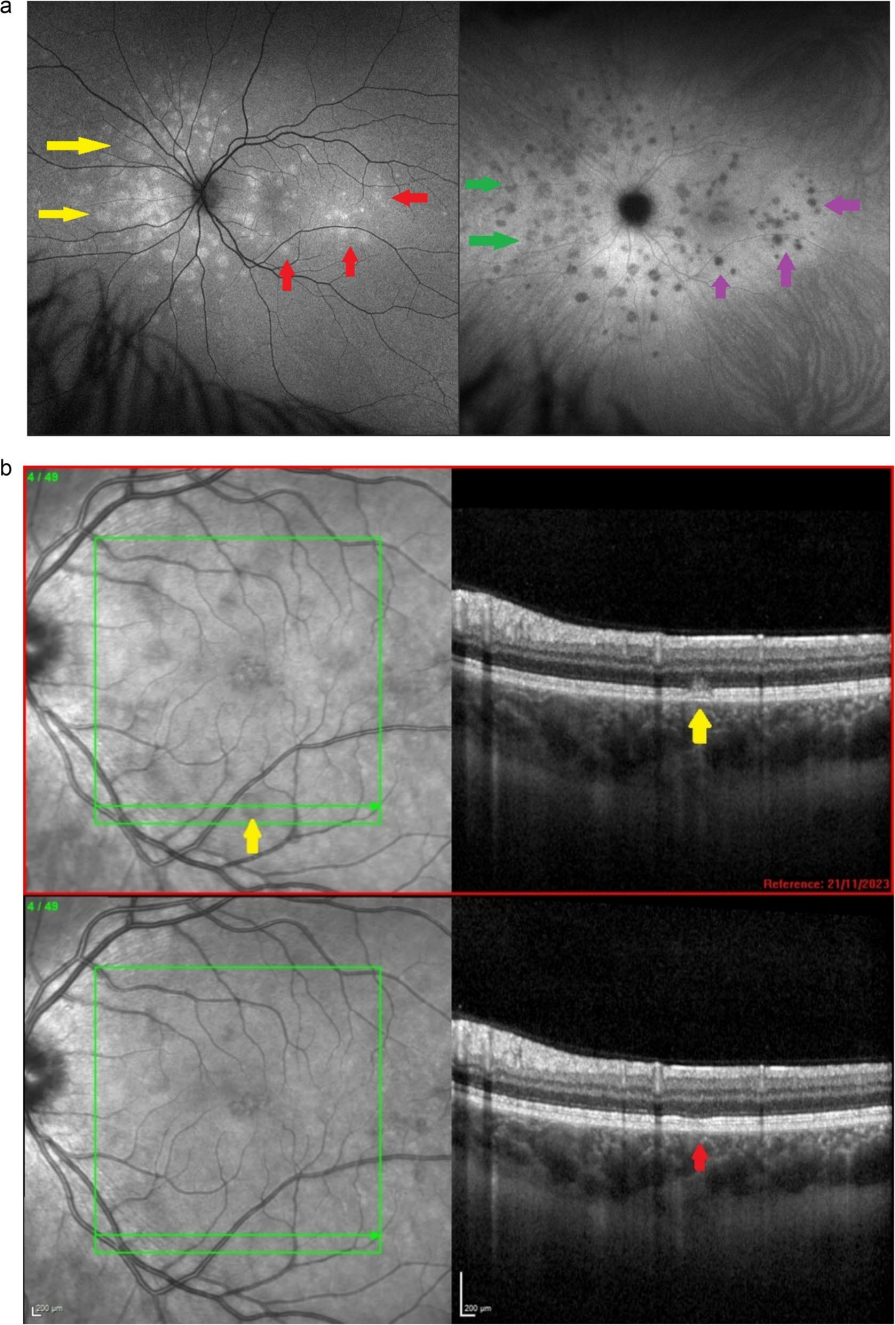
**a** Discrepancy between ICGA and FAF images indicating that end-choriocapillary non-perfusion is at the origine of photoreceptor damage. Left frame: autofluorescence of a patient diagnosed with MEWDS. The lesions nasally to the optic disc (yellow arrows) demonstrate established outer retinal damage (marked hyperautofluorescence) while the lesions temporal to macula (red arrows) are less marked. Comparing to the ICGA (right frame) it is noticed that the lesions nasally to the ON (green arrow) are less hypofluorescent (probably due to reperfusion) than the lesions temporal to the macula which are darkly hypofluorescent (crimson arrows). Probably the lesions nasally to ON are older lesions that already affected the outer retina as seen in FAF. The temporal lesions are fresher with the outer retina damage not yet completely established. This comparison can explain why MEWDS is a primary choriocapillaritis / choriocapillary vasculitis and not a photoreceptoritis as the hypoperfusion precedes the outer retina damage. **b** Comparison of SD-OCT at presentation (top image) and in 6 weeks follow-up (bottom image). OCT through one of the lesions (seen hypofluorescent in ICGA) showing hyperreflectivity and disruption of the IS/OS (yellow arrows, top picture). After 6 weeks, without any treatment, OCT showed improvement (red arrow, bottom picture)

This MEWDS case shows asymmetry of FAF and ICGA lesions suggesting that hyperautofluorescent areas seem to be maximal in areas where ICGA is comparatively less intense probably indicating re-perfusion whereas in other regions ICGA is intensively dark compared to less pronounced FAF hyperautofluorescence indicating that non-perfusion is preceding and present before consequent secondary damage to photoreceptor occurs (FAF-hyperautofluorescence) (Fig. 17a). SD-OCT at presentation passing through one of the darker lesions in ICGA showed hyperreflectivity of the outer segment and disruption of IS/OS (Fig. 17b).

##### Acute posterior multifocal placoid pigment epitheliopathy (APMPPE)

APMPPE also belongs to the group of primary choriocapillaritis / choriocapillary vasculitis entities. It is called primary because the trigger is also unknown. The name is a misnomer as the lesion process was attributed to the retinal pigment epithelium (RPE) when first described by Don Gass [18]. The pathophysiological explanation was reoriented, thanks to ICGA, towards the exact explanation of the disease process, namely inflammatory choriocapillaris vasculitic non-perfusion. Deutman had already understood the exact disease mechanism before the availability of ICGA. He had mentioned that in the early FA frames of APMPPE, there was perfusion delay of the choriocapillaris and he suggested the more appropriate denomination of the disease by calling it Acute Multifocal Ischaemic choriocapillaritis (AMIC) [13, 19] Indocyanine green angiography (ICGA) later confirmed the theory of choriocapillaris perfusion disturbance, showing that the perfusion delay described by Deutmann was not only a perfusion delay but choriocapillaris non-perfusion as the dark non-perfused areas remained until late angiographic frames [10].

**Fig. 18:**
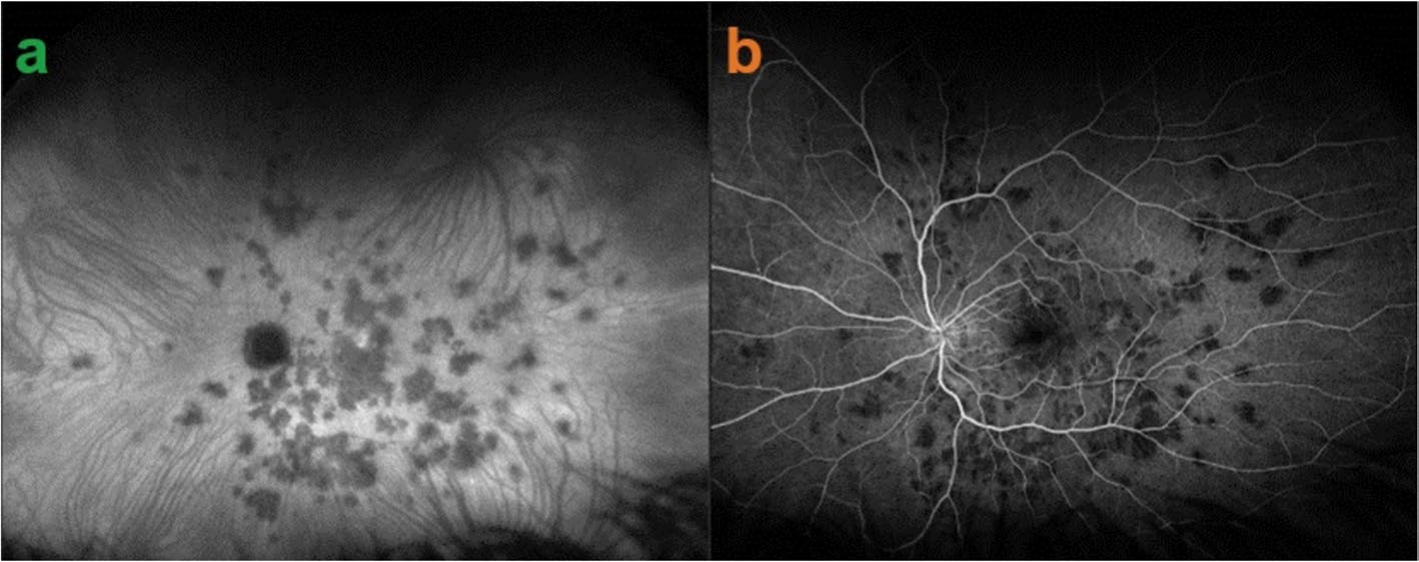
Correlation of late ICGA phases with early FA phases in APMPPE. **a** ICGA late phase demonstrated geographic area of non-perfusion of the choriocapillaris. The lesions correlate well with those seen in early stages of FA. **b** It is on these FA images that Deutman elaborated his correct interpretation of the disease process in APMPPE

In APMPPE/AMIC, inflammatory non-perfusion is bilateral and affects larger choriocapillaris or pre-choriocapillaris vessels than in MEWDS. Therefore, the areas of hypoperfusion are more extended and coalescent. It was considered to be a self-resolving condition, but later studies established that treatment with systemic corticosteroids was necessary, especially in cases with extended macular involvement [20]. APMPPE is a vasculitis affecting the choriocapillaris and is sometimes associated with cerebral vasculitis. ICGA can demonstrate choriocapillaris vasculitis as will be shown in the illustrative cases reported below. FA can demonstrate the choriocapillaris non-perfusion in the first 60 s of the angiogram which corresponds to the hypofluorescent areas seen on the ICGA up to late frames (Fig. 18).

**Case of APMPPE**

**Fig. 19:**
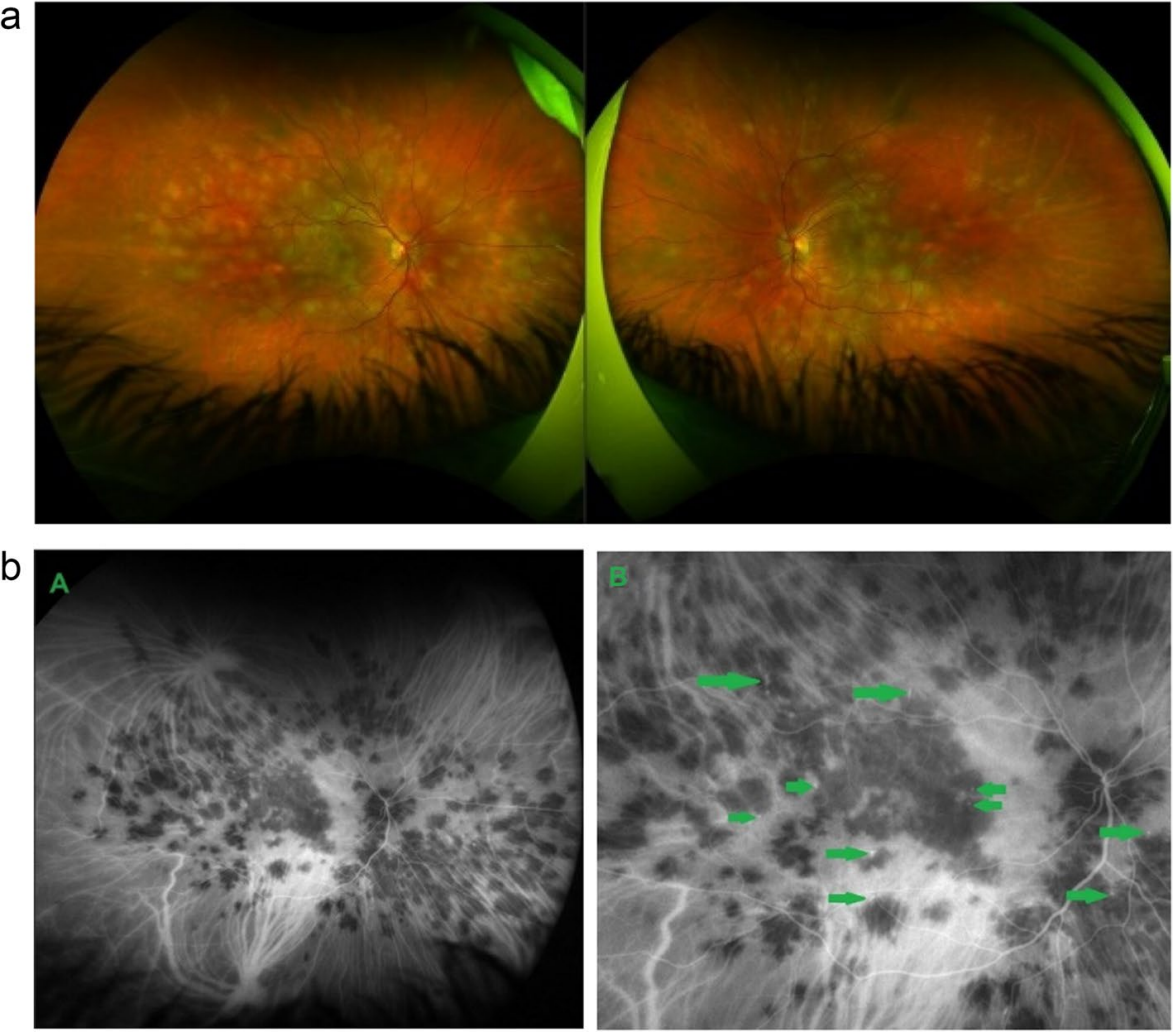
**a **Fundus appearance of APMPPE. Multiple bilateral placoid lesions.** b**/A. ICGA image of same patient as 19a. Numerous geographic areas of hypoperfusion. Note that the stromal vessels are minimally involved and can be well distinguished outside the choriocapillaritis APMPPE lesions (A general view; Intermediate phase of the ICGA of the RE of a patient with APMPPE. We note the hypofluorescent areas corresponding to the non-perfusion of the choriocapillaris. **b**/B. Same APMPPE patient as in 19a & 19b/A. B) Blow-up of part of the global view A, zoomed on the posterior pole. Green arrows pinpoint multiple hyperfluorescent spots or lines, adjacent to the areas of the hypoperfusion, showing vasculitis of the choriocapillaris vessel corresponding probably to the vasculitic occluded feeding vessel of the hypofluorescent dark lobules

A 25-year-old female patient presented with bilateral scotomas and vision loss. Fundus examination showed diffuse scattered white lesions in the posterior pole and mid-periphery. FA in the early angiographic phase and ICGA in the intermediate-late phases showed hypofluorescent areas of choriocapillaris non-perfusion (Fig. 19a).

This patient’s clinical case gave a very precious insight into the potential vascular pathophysiology of APMPPE as shown by ICGA (Fig. 19b/A). High magnification of ICGA angiographic frames of the intermediate phase showed that in the close vicinity adjacent to many hypofluorescent non-perfusion areas there were often punctiform or elongated hyperfluorescent structures probably indicating an inflamed vessel at the origin of the hypofluorescent areas of choriocapillaris non-perfusion (Green arrows in Fig. 19b/B).

**Case of APMPPE**

**Fig. 20:**
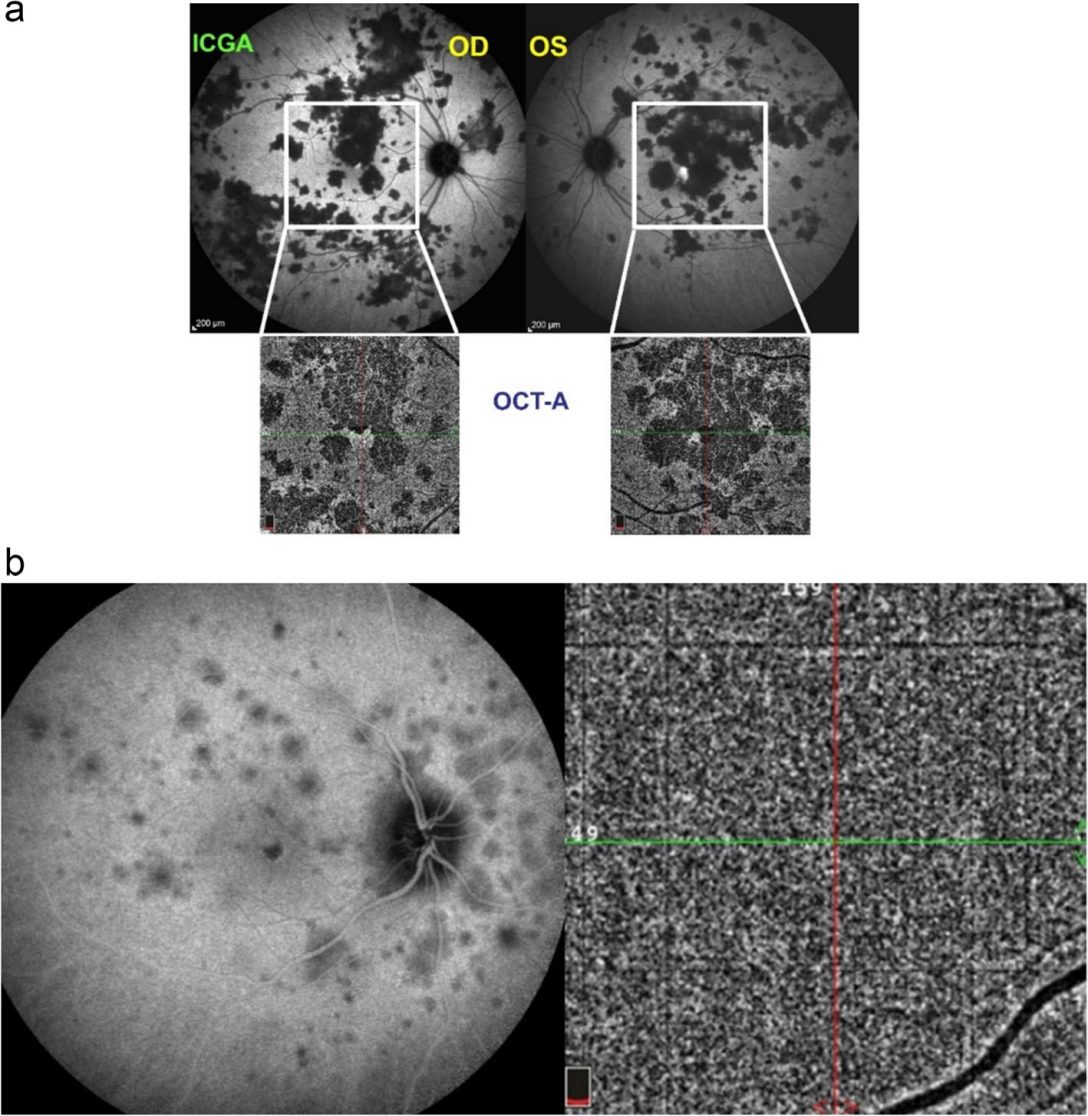
**a** APMPPE. Young male patient complaining of subjective scotomas and photopsias presenting widespread geographic and confluent areas of hypofluorescent non-perfused areas, precisely delineated on ICGA. The lesions in the posterior pole are also detected by OCT-A, showing the areas of choriocapillary drop-out. OCT-A is very useful for non-invasive follow-up of lesions, being however limited to the posterior pole. **b** MEWDS. Young male patient who consulted for unilateral photopsias and subjective scotomas. ICGA (left) shows numerous limited areas of end-choriocapillary non-perfusion that, in contrast to APMPPE (Fig. 20a) do not appear as drop-out areas on OCT-A (right) because OCT-A does not detect slow flow perfusion and therefore cannot show its drop-out

This 28-year-old patient consulted for bilateral photopsias and subjective scotomas. Fundus examination showed multiple posterior pole and mid-periphery placoid lesions. On ICGA these lesions corresponded to numerous sometimes coalescent dark areas of choriocapillaris non-perfusion indicating larger vessel involvement. OCT-A showed large areas of choriocapillary drop out with absence of flow (Fig. 20a). This is in contrast with MEWDS, where circulatory impairment involves end-capillary choriocapillaris with reduced flow that cannot be detected by OCT-A. Consequently OCT-A is unable to determine whether there is end-choriocapillary closure and complete absence of flow which can only be detected by ICGA (Fig. 20b).

##### Idiopathic Multifocal Choroiditis (MFC)

**Fig. 21:**
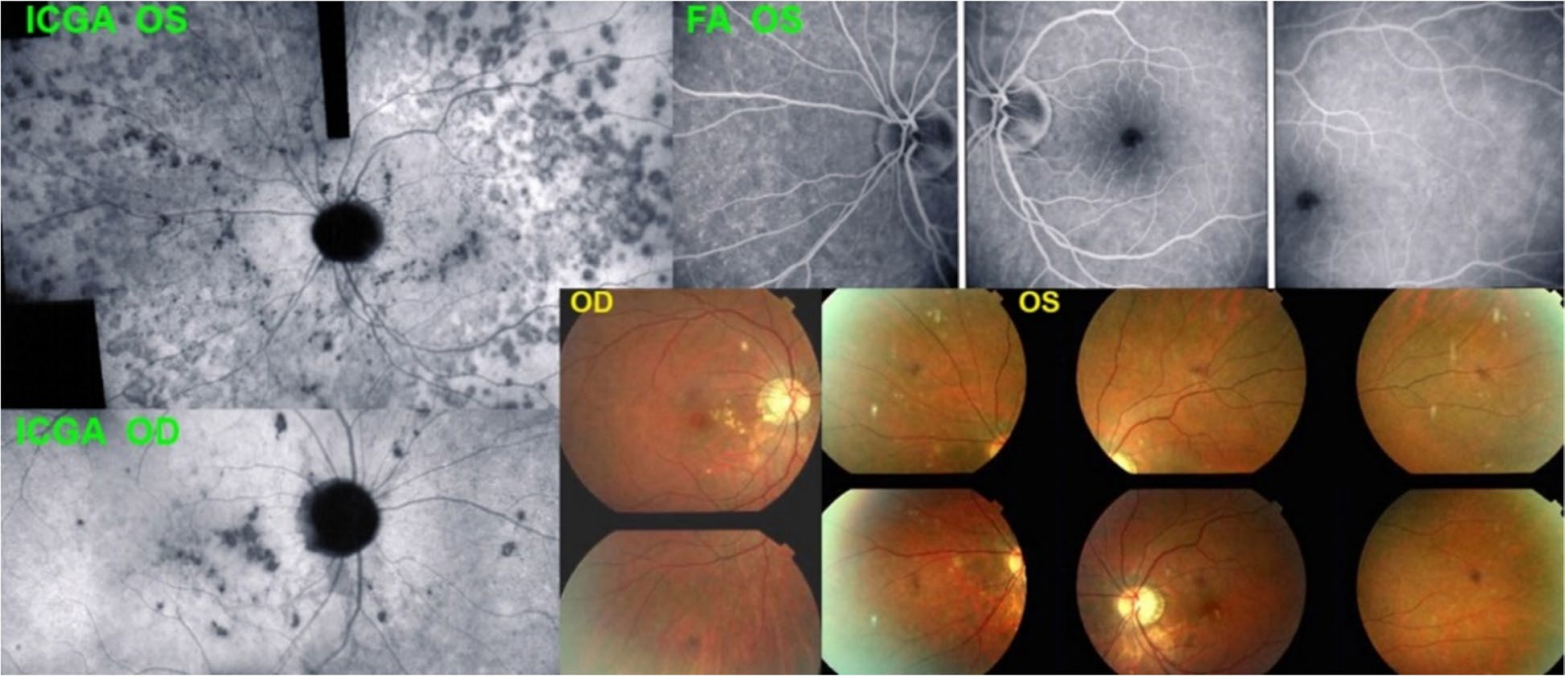
Multifocal choroiditis (MFC). Case of bilateral MFC, active in the left eye (top left) and inactive in the right eye (bottom left) with only chorioretinal scars but no vasculitis of the choriocapillaris. Fundus pictures (bottom right) show bilateral scars but do not allow to determine whether the disease is active. The widespread occult choriocapillaris vasculitis is shown by ICGA (top left), which is absolutely not suspected on FA (3 top right frames)

The vasculitic process in MFC is comparable to that of MEWDS. Indeed, it is not rare to make the diagnosis of MEWDS at the time of the first episode of MFC. The findings are absolutely similar to MEWDS (Fig. 21). It is only when a second inflammatory episode occurs that the diagnosis has to be rectified. MFC is a recurrent disease ultimately affecting both eyes and characterized by chorioretinal scars, which is not the case of MEWDS that is unilateral, usually limited to one episode and does not produce scars. The severity of MFC is very diverse but the inflammatory non-perfusion of the choriocapillaris is often prolonged and widespread, the consequent ischemia being at the origin of the scars and of choroidal neovascular membranes that develop in up to 30% of cases [21]. MFC very often needs aggressive immunosuppressive treatment [21].

##### Serpiginous choroiditis (SC)

SC is the most severe form of choriocapillaritis involving larger and progressing areas of non-perfusion probably due to vasculitis of larger choriocapillaris vessels or pre-choriocapillary arterioles [22]. One possible trigger of SC is due to Mycobacterium tuberculosis which has to be excluded by performing an interferon-gamma release assay (IGRA). In case of IGRA negativity the condition is called idiopathic SC which needs dual to triple immunosuppressive treatments to halt the progression of the disease.

**Illustrative case of idiopathic SC**

**Fig. 22:**
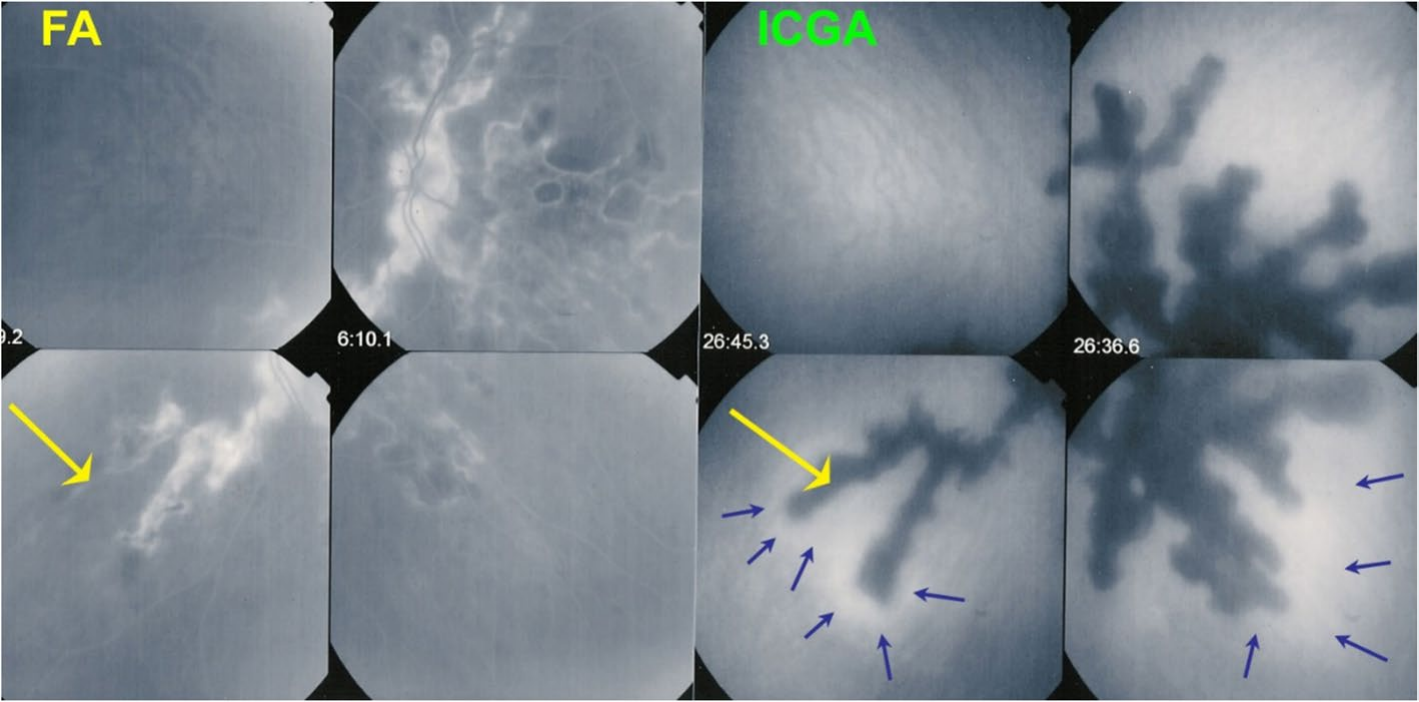
Serpiginous choroiditis. Non-perfusion in SC involves larger choriocapillaris vessels and produces extended areas of non-perfusion leading to chorioretinal atrophy if treatment is not initiated promptly. ICGA clearly delineates atrophic in addition to non-perfused areas (yellow arrow on ICGA frame), whereas FA only shows atrophic areas but not non-perfused areas (yellow arrow on FA frame). In addition, ICGA shows perilesional hyperfluorescence (dark blue arrows) indicating possible disease activity

This 64-year-old patient had been treated on and off with systemic corticosteroids for several years with, however regular progression of the serpiginous lesions (Fig. 22). An IGRA test was negative, indicating that the patient had never been exposed to Mycobacterium tuberculosis, the diagnosis being thus the idiopathic form of SC. The initiation of a combined immunosuppressive therapy including systemic corticosteroids, azathioprine and cyclosporin allowed to halt the progression of the disease.

#### Primary stromal choroidal vasculitis

The most characteristic sign of stromal choroiditis on ICGA is the presence of HDDs. These choroidal foci represent an inflammatory process taking its origin within the choroidal stroma which, logically, generates and is accompanied by inflammation of the stromal choroidal vessels. In contrast to choriocapillaris vessels which are fenestrated, these larger stromal vessels are impermeable in a normal non-inflammatory situation. In case of choroiditis these vessels become leaky and allow ICG to egress rendering the vascular pattern fuzzy in the intermediate angiographic phase and does not allow to see the normal course of the choroidal vessels in the late phase because of the abnormal ICG coming from inflamed larger vessels, adding to the physiological egression of ICG from the choriocapillaris fenestrations causes late diffuse hyperfluorescence [23]. Because the lesion process is originating from within the choroid, involvement is quite uniform in all the fundus areas with evenly distributed HDDs and diffuse hyperfluorescence. Vogt-Koyanagi Harada disease (VKH), Sympathetic Ophthalmia (SO) and HLA-A29 Birdshot Retinochoroiditis (BRC) are considered as primary stromal choroiditis entities because the inflammatory process starts from within the choroidal stroma.

##### Choroidal stromal vasculitis in VKH disease

**Fig. 23:**
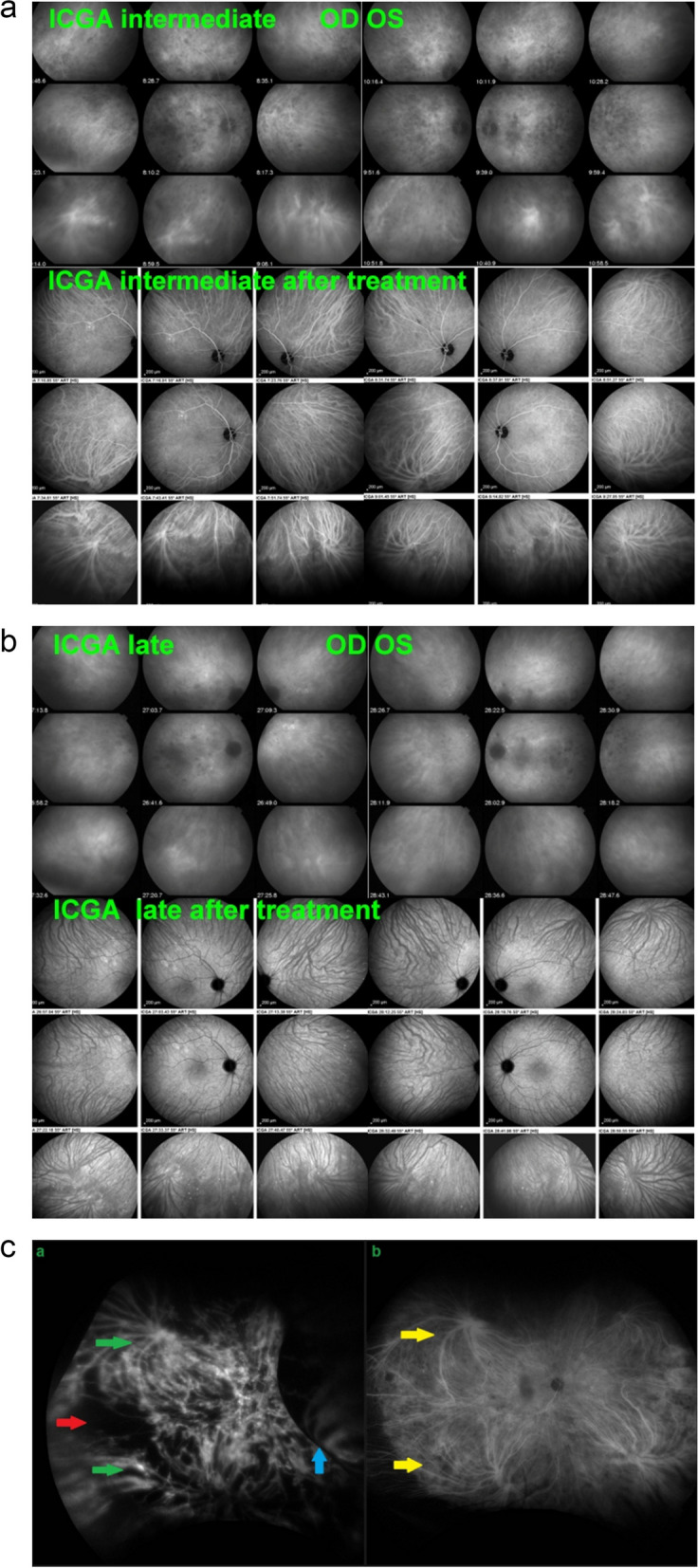
**a** Choroidal vasculitis in VKH disease, intermediate angiographic phase. Pronounced choroidal vasculitis is present overshadowing HDDs barely detectable (top 2 sets of 9 frames). After treatment (bottom 2 sets of 9 frames) the normal pattern of choroidal vessels are again clearly identifiable with resolution of HDDs. **b** Choroidal (ICGA) vasculitis in VKH disease, late angiographic phase. Pronounced choroidal vasculitis with late hyperfluorescence obscuring the normal pattern of choroidal vasculature (top 2 sets of 9 frames). After treatment, the normal vascular pattern is again identifiable, in negative, as there is no ICG dye any longer within the circulation (bottom 2 sets of 9 frames). **c **VKH ICGA mid-phase (10’). **c**/a) At presentation, we notice marked choroidal vasculitis (green arrows) as well as choroidal ischemia (red arrow). The inflammation was such that the patient developed choroidal detachment (blue arrow). **c**/b) After treatment there is improvement of the choroidal vasculitis (yellow arrows) and reperfusion of the choroid

**Fig. 24:**
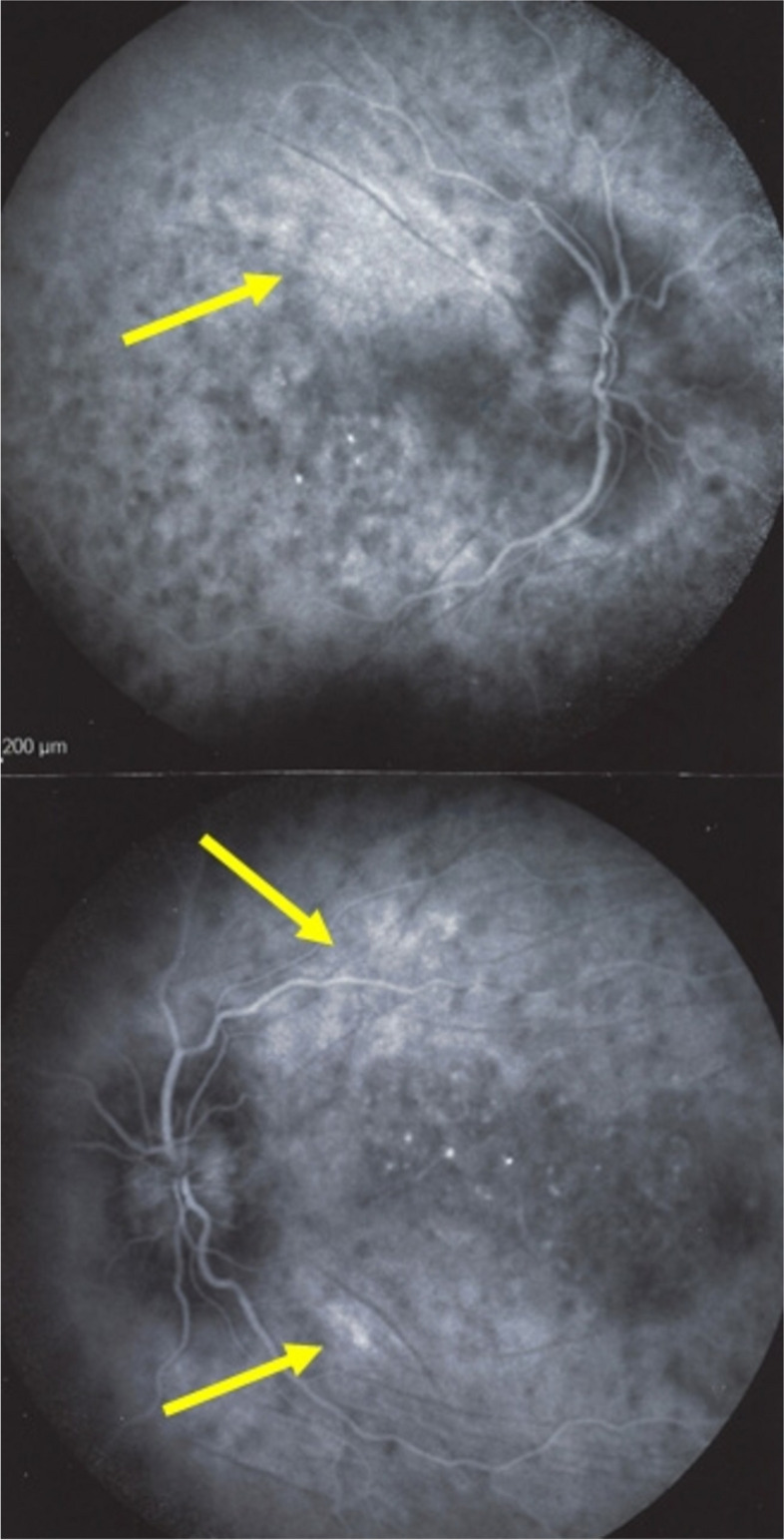
Choroidal vasculitis in VKH disease. Early angiographic phase showing early hyperfluorescent vessels (arrows) and ICGA disc hyperfluorescence only seen on ICGA in hyperacute inflammation

**Fig. 25:**
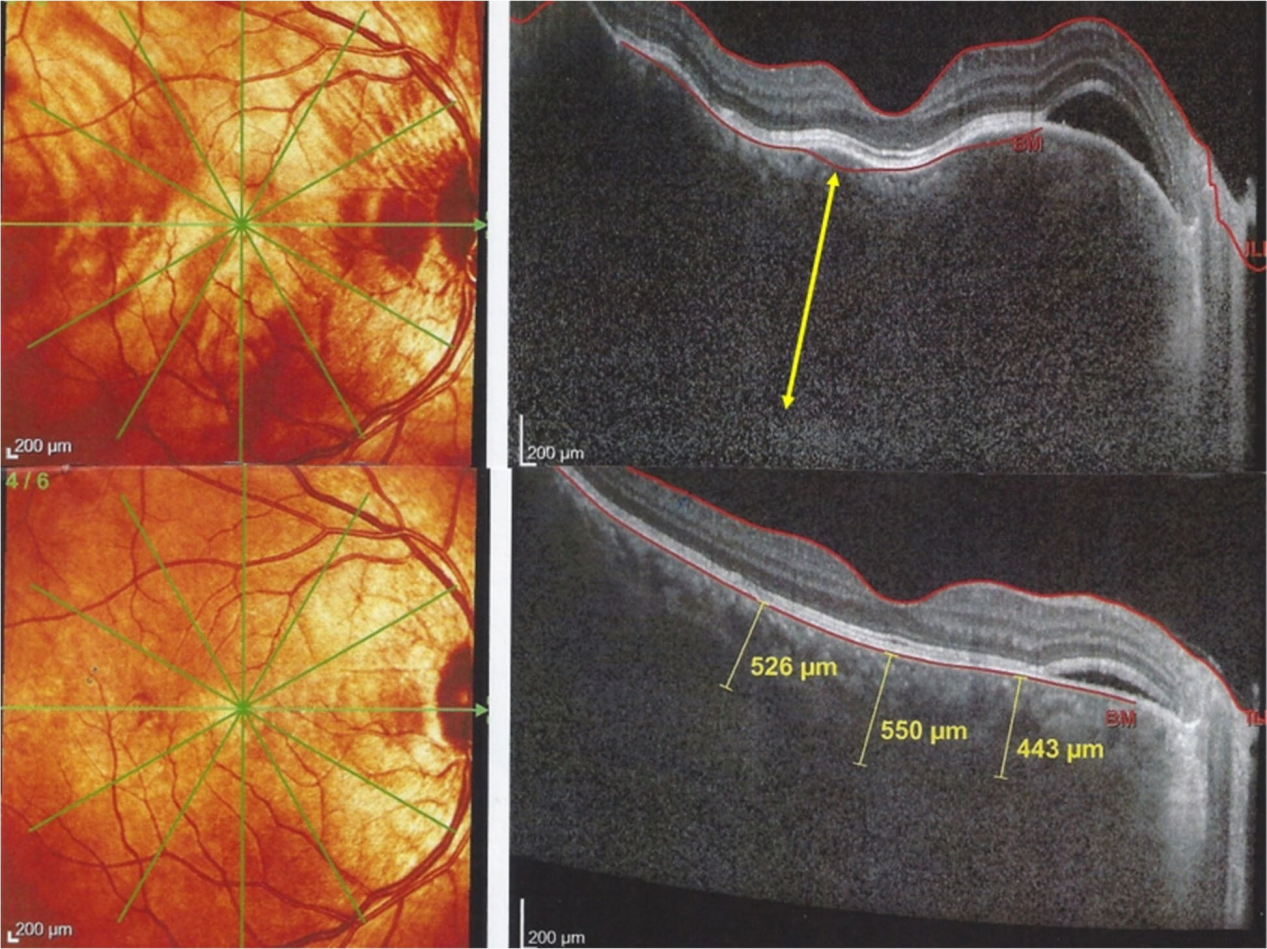
Choroidal vasculitis in VKH disease, EDI-OCT thickening of the choroid. At the early stage of VKH, there is pronounced choroidal thickening barely measurable (top image), gradually returning to normal thickness after treatment (bottom image)

In VKH, histopathologic evaluation reveals thickening of the choroid with stromal cellular inflammation constituted by macrophages, lymphocytes, and epithelioid cells containing melanin, as the consequence of an autoimmune process directed against melanin-associated proteins within choroidal stromal pigmented islets. The presence of evenly sized and evenly distributed HDDs is the characteristic finding by ICGA in VKH. The process is at first limited to the choroidal stroma before spilling over secondarily to neighbouring structures such as the retina and optic disc. VKH can therefore be considered as a purely stromal choroiditis, as inflammation starts exclusively in the choroidal stroma [24]. The consequence of the choroidal autoimmune infiltration is the presence of choroidal vasculitis evidenced by ICGA and characterised by individualised early hyperfluorescent choroidal vessels, general staining of large choroidal stromal vessels making them appear fuzzy in the intermediate angiographic phase followed by diffuse hyperfluorescence that overshadows HDDs less well discernible in the late phase. Other ICGA signs include disc hyperfluorescence rarely seen on ICGA unless choroidal inflammation is pronounced which is the case in VKH and ICGA hyperfluorescent pinpoints marking points of leakage from the choroid at the origin of the serous retinal detachments (Figs. 23, 24 and 25).

##### Choroidal stromal vasculitis in Sympathetic Ophthalmia

**Fig. 26:**
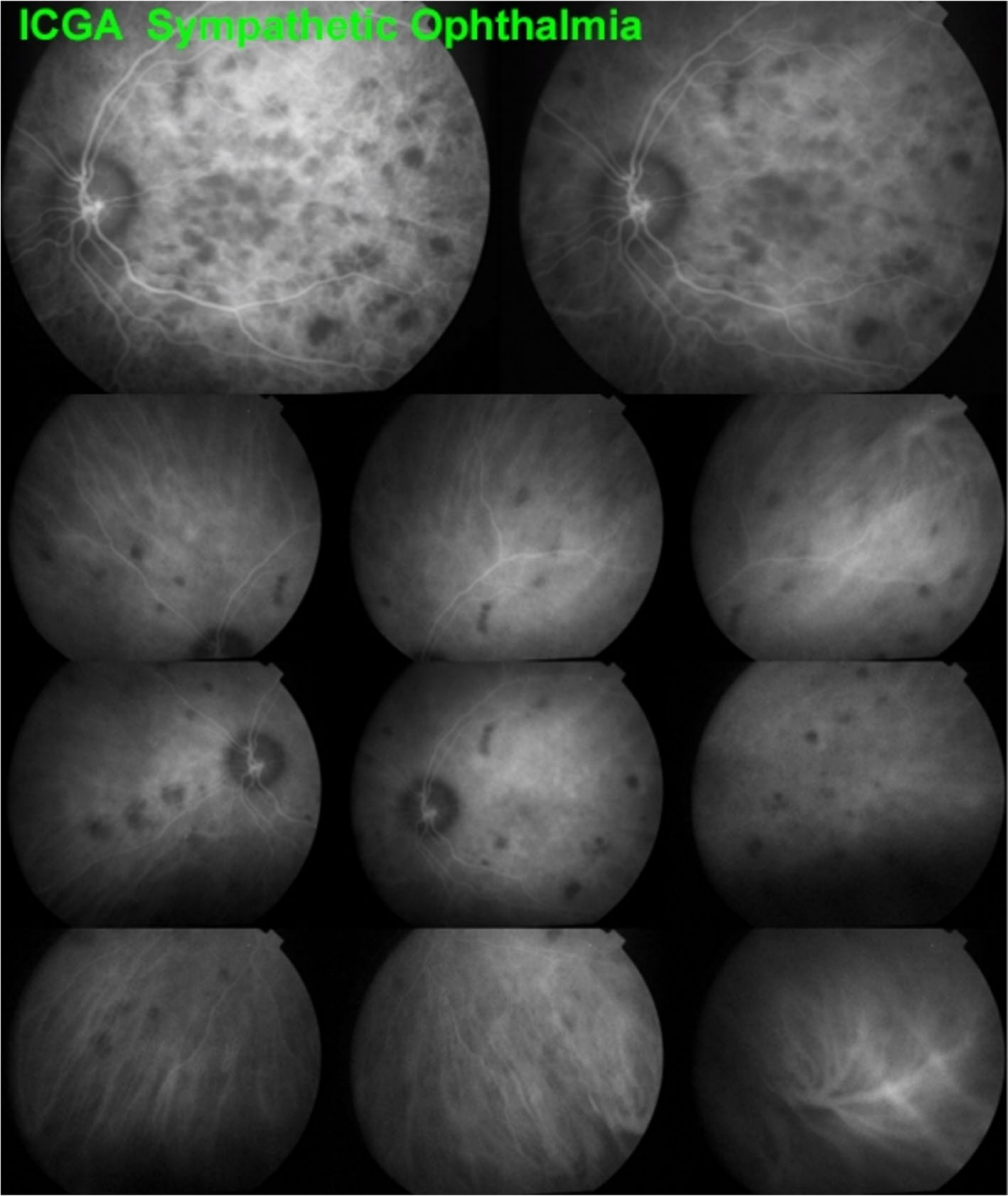
Stromal vasculitis in Sympathetic Ophthalmia. This 24-year-old Indian lady had undergone vitrectomy followed by cataract operation and filtering surgery in her right eye.Two months after the last surgery VA in her left eye decreased and laser flare photometry indicated subclinical anterior chamber inflammation with flare amounting to18.2 ph/ms. Intermediate phase ICGA showed numerous HDDs and choroidal stromal vasculitis (top 2 frames). Late panorama frames (bottom 9 frames) showed substantial choroidal stromal vasculitis with persistent HDDs and some evanescent HDDs in the posterior pole indicating partial thickness foci. All angiographic signs resolved after introduction of systemic corticosteroid, Mycophenolate and Infliximab therapy

The disease process is similar to VKH. The autoimmune reaction is thought to be triggered by the exciting eye that has suffered a penetrating injury or multiple intraocular surgeries. Therefore, the angiographic findings are comparable to VKH disease, being usually, however, less severe [25] (Fig. 26).

##### Choroidal stromal vasculitis in HLA-A29 Birdshot retinochoroiditis (BRC)

**Fig. 27:**
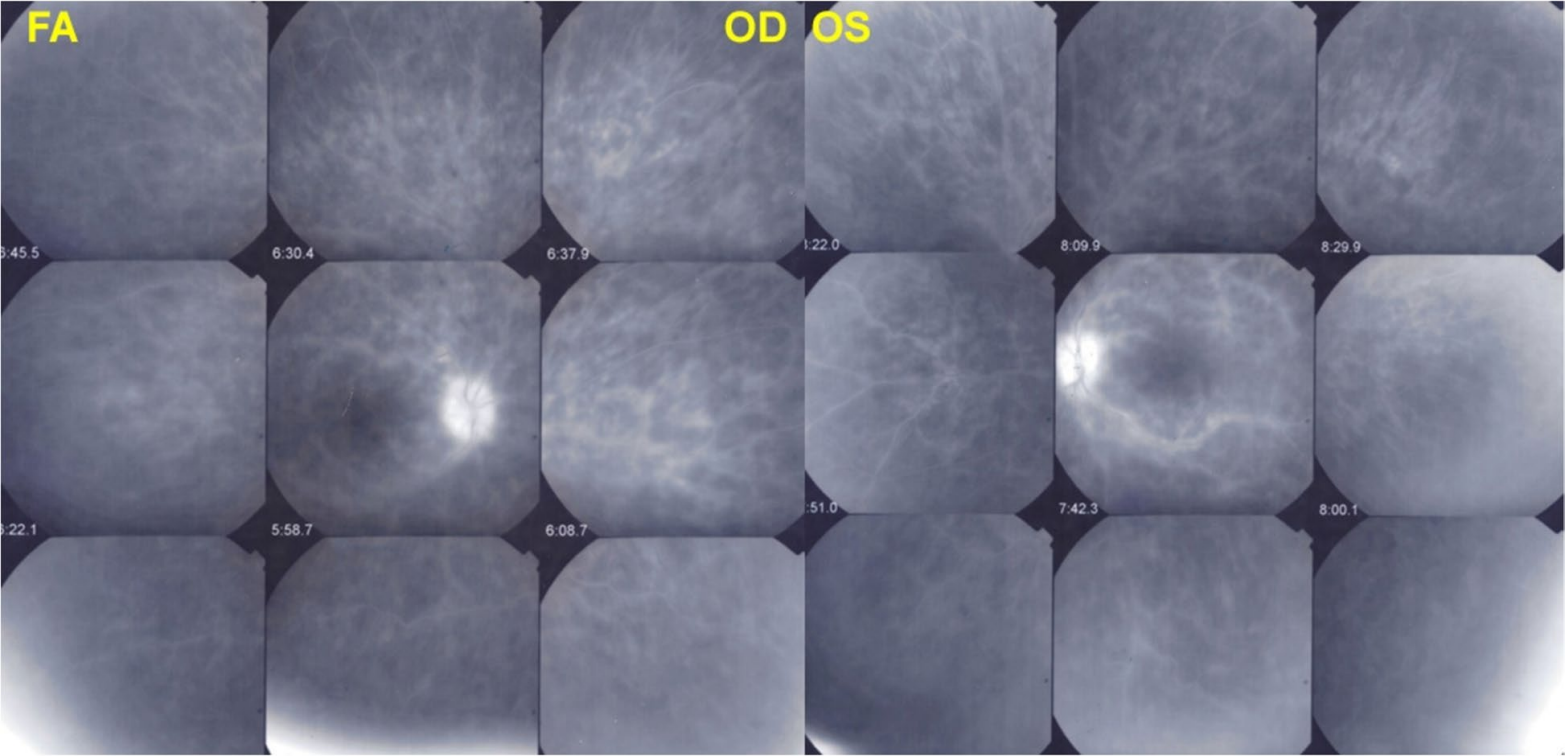
Retinal involvement in BRC. Retinal vasculitis is a constant finding in BRC and develops concomitantly to stromal choroiditis. Diffuse bilateral retinal vasculitis is present in 100% of BRC and is an early occurrence. It is characterised by a very leaky vasculitis of small and large vessels with profuse exudation, macular oedema often sparing the fovea, thick FA sheathing/staining of large posterior pole vessels, arterio-venous circulatory pseudo-delay, and pronounced disc hyperfluorescence

**Fig. 28:**
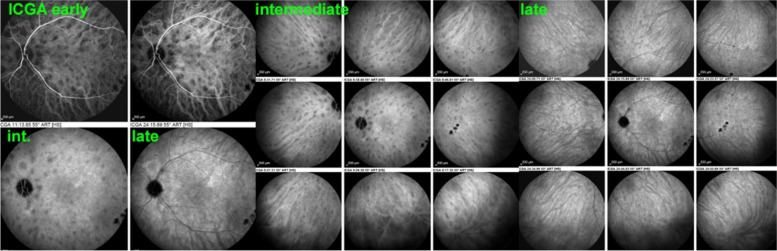
BRC choroidal vasculitis in a case of moderate severity. Posterior pole involvement is seen in the left quartet of frames (left), in the intermediate and late frames, normal choroidal vasculature is less well recognizable; note that HDDs are fading away on the late frame, indicating partial thickness of choroidal infiltrates. On panfundal intermediate and late frames (middle and right sets of 9 frames) peripheral choroidal vasculitis is minimal, as both intermediate and late angiographic phases, the normal vascular pattern is identifiable. Note the fading away of HDDs in the late angiographic phase (right set of 9 frames), indicating partial thickness lesions

**Fig. 29:**
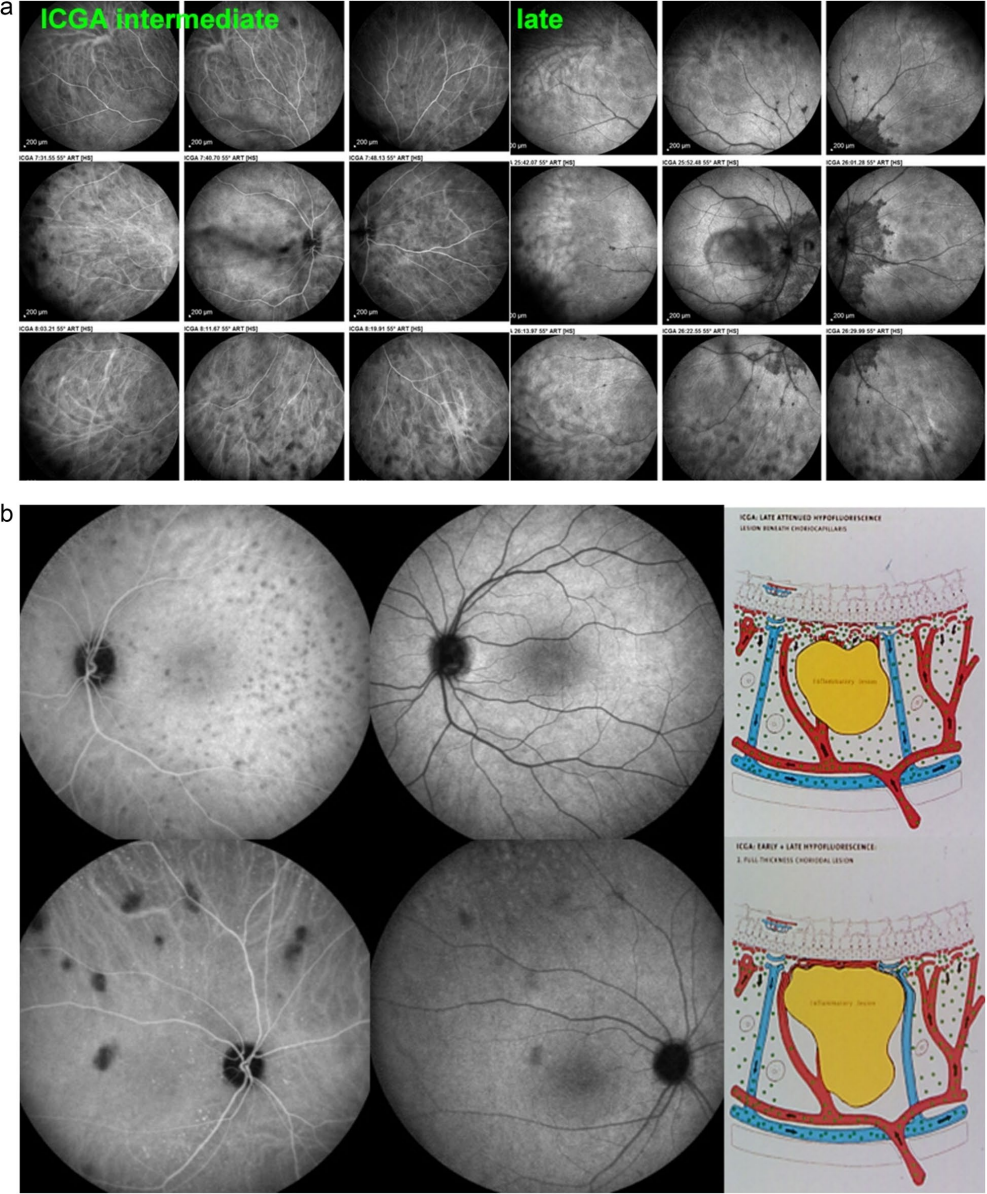
**a** BRC choroidal vasculitis in a case of pronounced severity.Numerous HDDs are seen in the intermediate phase (left set of 9 frames) remaining until late phase (right 9 frames). However, choroidal stromal vasculitis is usually always less than in VKH disease, as choroidal vessel pattern is still identifiable in both intermediate and late phases. **b** Partial thickness HDDs. Top 2 frames and corresponding cartoon on right: In the intermediate angiographic phase (top left) of this BRC case, numerous HDDs that fade away in the late angiographic frame (top middle), corresponding to partial thickness lesion as illustrated on cartoon on the right. Bottom 2 frames and corresponding cartoon on the right: HDDs visible in the intermediate angiographic phase (bottom left) still visible in the late angiographic frame (bottom middle) corresponding to full thickness lesion as illustrated on the corresponding cartoon on the right

BRC is a presumptive autoimmune condition involving both the retinal and the choroidal compartments. Unlike in VKH disease which is a purely stromal choroiditis, in BRC inflammation develops concomitantly in parallel in the retina showing a pathognomonic vasculitis (Fig. 27) and in the choroid which distinguishes it from VKH disease [26]. Nevertheless, the choroidal involvement is a genuine stromal choroiditis (Figs. 28 and 29a). Thus, ICGA signs of vasculitis in BRC are comparable to VKH disease, although less severe. Early hyperfluorescent vessels and disc hyperfluorescence are rarely seen. As for VKH disease the presence of HDDs is a prominent feature associated with choroidal vasculitis. However choroidal infiltration is often of partial thickness explaining that in the late ICGA angiographic frames HDDs have a tendency to fade (Fig. 29b).

### Secondary choroidal vasculitis

#### Secondary choriocapillary vasculitis / choriocapillaritis

##### Acute Syphilitic Posterior Placoid Chorioretinitis (ASPPC)

Acute Syphilitic Posterior Placoid Chorioretinitis (ASPPC) is one of the disease expressions caused by ocular syphilis. It is not a direct infectious process but an immunologic reaction triggered by Treponema Pallidum causing secondary non-perfusion of the choriocapillaris [27–29]. It has been shown that such an expression of syphilitic eye disease can be suppressed by corticosteroid therapy indicating the immunologic component of this condition [30]. However, a definitive cure of the disease can only be achieved by appropriate antibiotic therapy.

**Case report illustrating choriocapillaris non-perfusion in ASPPC**

**Fig. 30:**
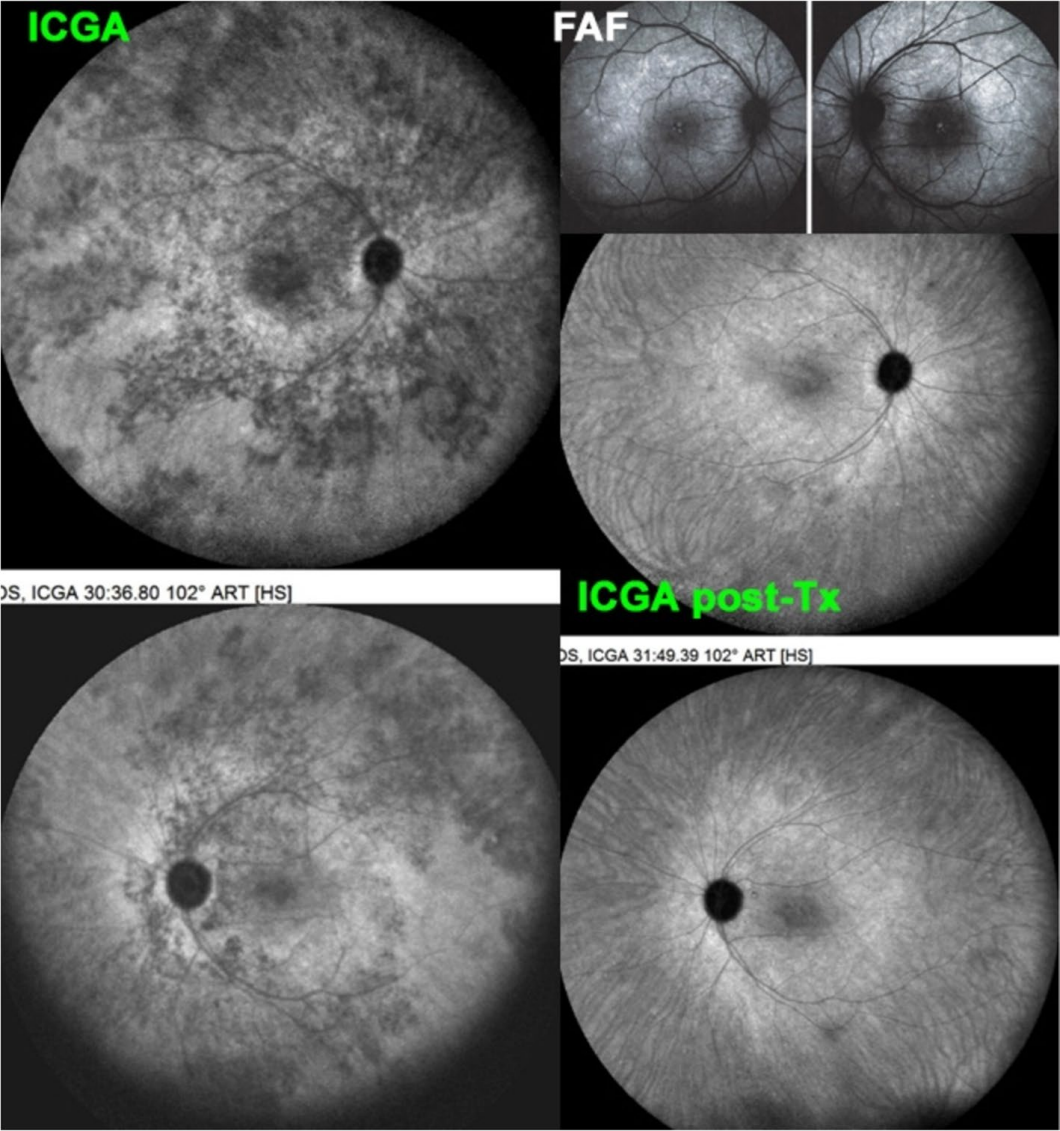
Acute Syphilitic Posterior Placoid Chorioretinitis (ASPPC). At presentation (2 left frames) numerous geographic areas of hypofluorescence are present on ICGA delineating the extensive choriocapillaris vasculitis producing choriocapillaris non-perfusion, which is co-localized with fundus hyperautofluorescence (FAF, right top 2 frames) indicating photoreceptor outer segment loss. On the middle and bottom left 2 frames all the non-perfused areas have recovered after treatment

This 27-year-old lady had been suffering from angina which was followed by a decrease of visual function in form of dim vision and subjective scotomas. At presentation visual acuity was reduced to 0.6 RE and 0.5 LE. There was subclinical anterior chamber inflammation detected by laser flare photometry amounting to 14.7 ph/ms OD and 8.3 ph/ms OS (normal values = 4–6 ph/ms). OCT showed bilateral cystoid macular oedema and extended areas of loss of outer photoreceptor segments corresponding to areas of hyperautofluorescence on fundus autofluorescence frames. FA showed bilateral retinal vasculitis, cystoid macular oedema and disc hyperfluorescence. ICGA showed extensive areas of geographic hypofluorescence indicating widespread choriocapillaris non-perfusion. Syphilis serology yielded an elevated VDRL test and elevated TPHA (treponema pallidum hemagglutination assay) serology. After antibiotic treatment (Ceftriaxone 2000 mg/day IM for 14 days) all the hypofluorescent areas of non-perfusion were again re-perfused (Fig. 30).

##### Tuberculosis related serpiginous choroiditis

Similar to syphilis, tuberculous ocular involvement can be twofold. It can take the form of direct infectious chorioretinitis or can present as a predominantly immune process causing tuberculosis-related multifocal/serpiginous choroiditis. In all forms of multifocal/serpiginous cases an Interferon-Gamma- Release Assay (IGRA) should be performed. In case the IGRA test is positive, the condition should be considered as a tuberculosis related immune form of serpiginous to be distinguished from idiopathic serpiginous choroiditis [31]. As for ASPPC, the mechanism consists of an immune-mediated choriocapillaris non-perfusion. The level of involvement in the vascular tree is more proximal than other choriocapillaris entities and involves larger vessels, producing extended ischemia. It is non-reversible and leaves choriorertinal scars if not treated in time. Adequate treatment consists of concomitant dual multiple anti-tuberculous antibiotic treatment and multiple immunosuppressants.

**Illustrative case of choriocapillary vasculitis/ choriocapillaritis in tuberculosis-related serpiginous choroiditis**

**Fig. 31:**
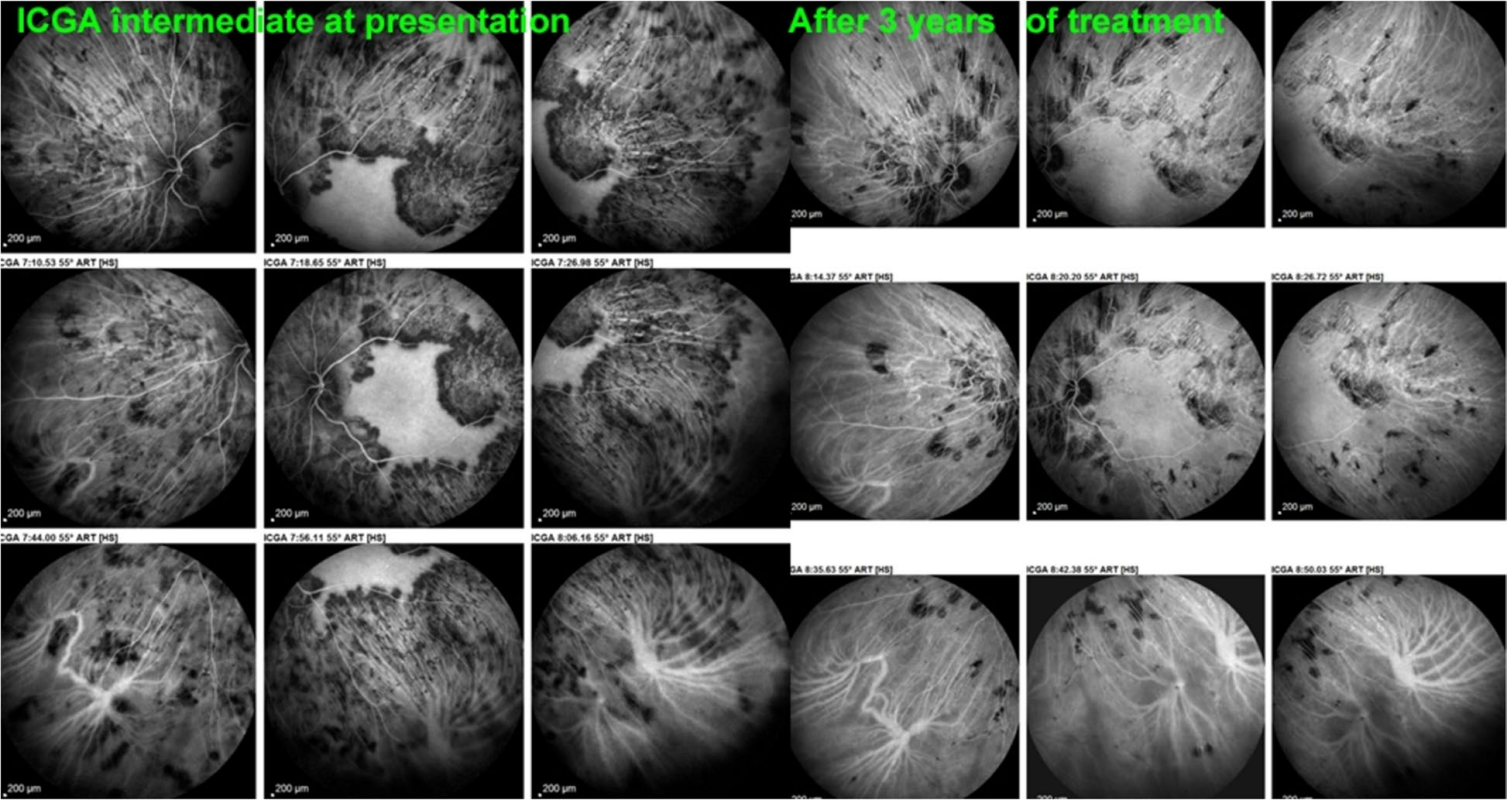
Tuberculosis-related Serpiginous choroiditis (choriocapillary vasculitis/ choriocapillaritis; intermediate angiographic phase (left eye). At presentation, widespread area of vasculitic choriocapillaris non-perfusion extended over the whole fundus, only sparing the central macular area showing normal background fluorescence (left 9 frames). After 3 years of combine antibiotic and immunosuppressive treatment (right 9 frames) most of the choriocapillaris non-perfusion has recovered. Only limited scattered areas of hypofluorescence remain representing chorioretinal scars

**Fig. 32:**
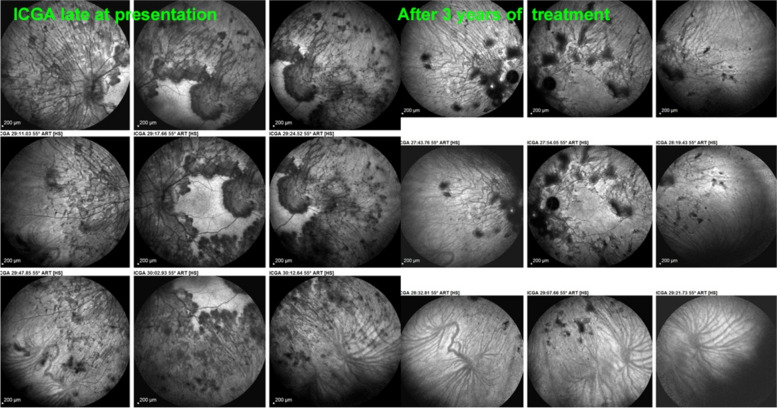
Tuberculosis-related Serpiginous choroiditis (choriocapillary vasculitis/ choriocapillaritis; late angiographic phase (left eye). The late ICGA angiographic frames show that numerous hypofluorescent areas are still present in the late angiographic phase indicating non-perfusion (left 9 frames). After treatment (right 9 frames) the scared areas are better visible and slightly more extended than on the intermediate ICGA frames

This 35-year-old male patient had been treated for a bilateral chorioretinitis on and off systemic corticosteroids and on and off anti-tuberculous antibiotics because of a positive IGRA test. These treatments were, however, never given concomitantly. At presentation in our centre, ICGA angiography showed vast areas of choriocapillaris non-perfusion. Prolonged treatment with quadruple anti-tuberculous drugs, including rifampicin, Isoniazid and Pyrazinamide (13 months) and Ethambutol (4 months) was given along with Prednisone starting with 100 mg/day, tapered to 0 after 36 months, cyclosporine starting with 700 mg/day (5.83 mg/kg) tapered to 0 after 36 months and infliximab 5 mg/kg every 4 weeks with interval increase to 6 weeks during 9 months, to 10 weeks during 11 months and discontinuation after 29 months. At the end of treatment, 36 months later, vast areas of non-perfused choriocapillaris having recovered were associated with areas of chorioretinal atrophy (Figs. 31 and 32).

**Choriocapillary vasculitis/ choriocapillaritis in tuberculosis-related serpiginous choroiditis**

**Fig. 33:**
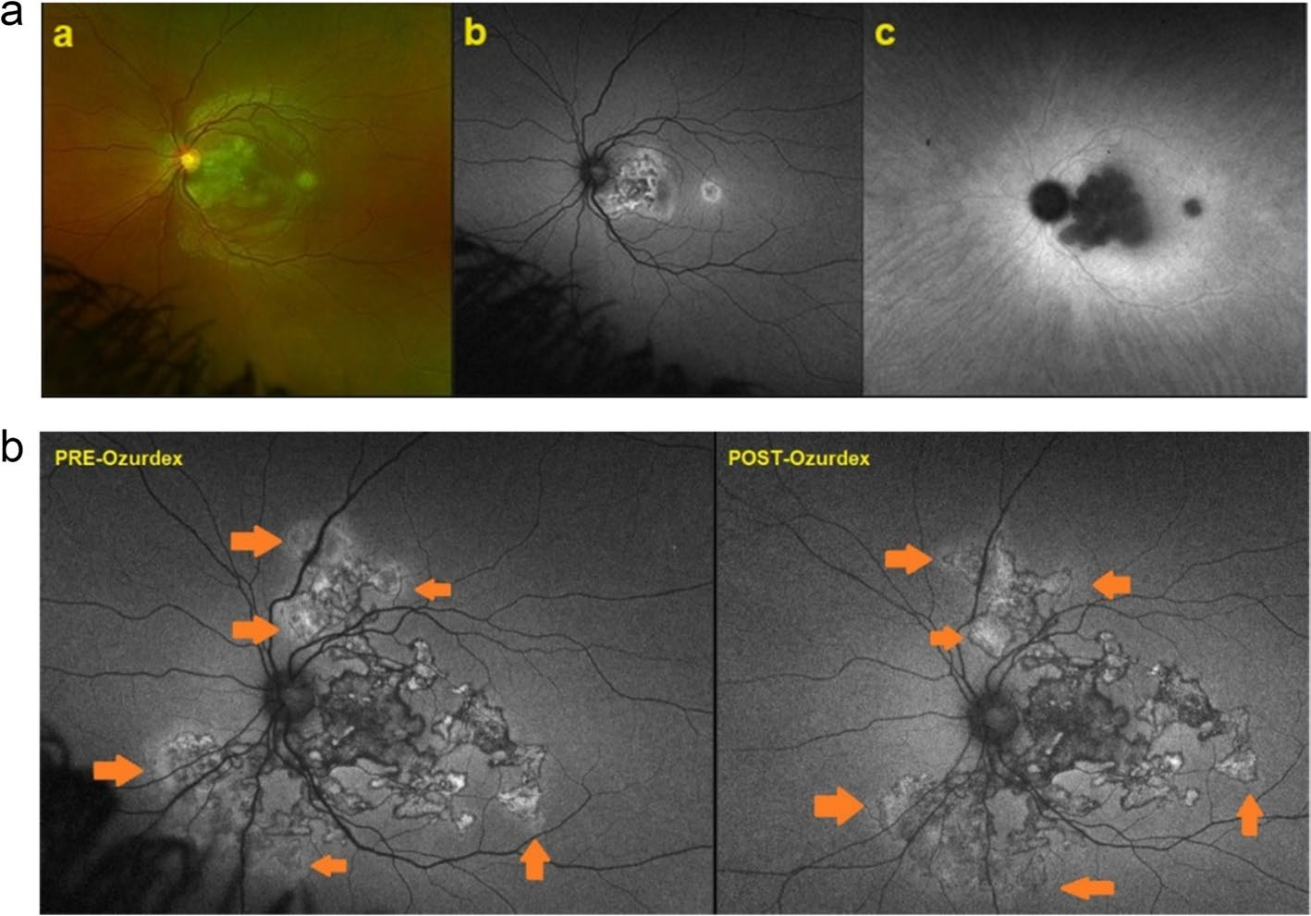
**a **Composition of fundus (left), FAF (middle) and ICGA (right) of a patient with TB - related SC. The fundus (**a**) reveals presence of whitish lesions while FAF (**b**) showed mixed hyperautofluorescence and hypofluorescence. ICGA (**c**) showed hypofluorescent areas due to choriocapillaris non perfusion and fuzziness of the choroidal vessels as well as hyperfluorescence surrounding the area of non-perfusion due to reactive choroidal vasculitis indicating disease activity. **b **FAF evolution of a TB related Serpiginous Choroiditis.Pre-Ozurdex® (left frame), still areas of activity visible, appearing hyperautofluorescent adjacent to established scars (arrows). Post-ozurdex® (right frame): the hyperautofluorescent lesions resolved and no new scars identified

A 22-year-old African man consulted for a vision loss in his left eye. Fundus examination revealed the presence of a whitish area between the optic disc and the macula as well as an isolated lesion temporal to the macula. Autofluorescence revealed the presence of hyperautofluorescence of the isolated lesion and mixed hyper-hypoautofluorescence of the larger lesion. ICGA revealed choriocapillaris non-perfusion and fuzziness of the choroidal vessels around the lesions (Fig. 33a).

Treatment with a high dose of steroids and ATT (anti-tuberculous treatment) was started. Due to pulmonary involvement, the start of second line immunosuppression was not permitted by the chest physicians. The evolution of the lesions continued as the patient was not compliant with treatment. Finally, he accepted the injection of a dexamethasone intravitreal implant (Ozurdex®) associated with oral steroids and quadruple antituberculous antibiotic treatment which stopped the disease progression (Fig. 33b).

#### Secondary stromal vasculitis

**Fig. 34:**
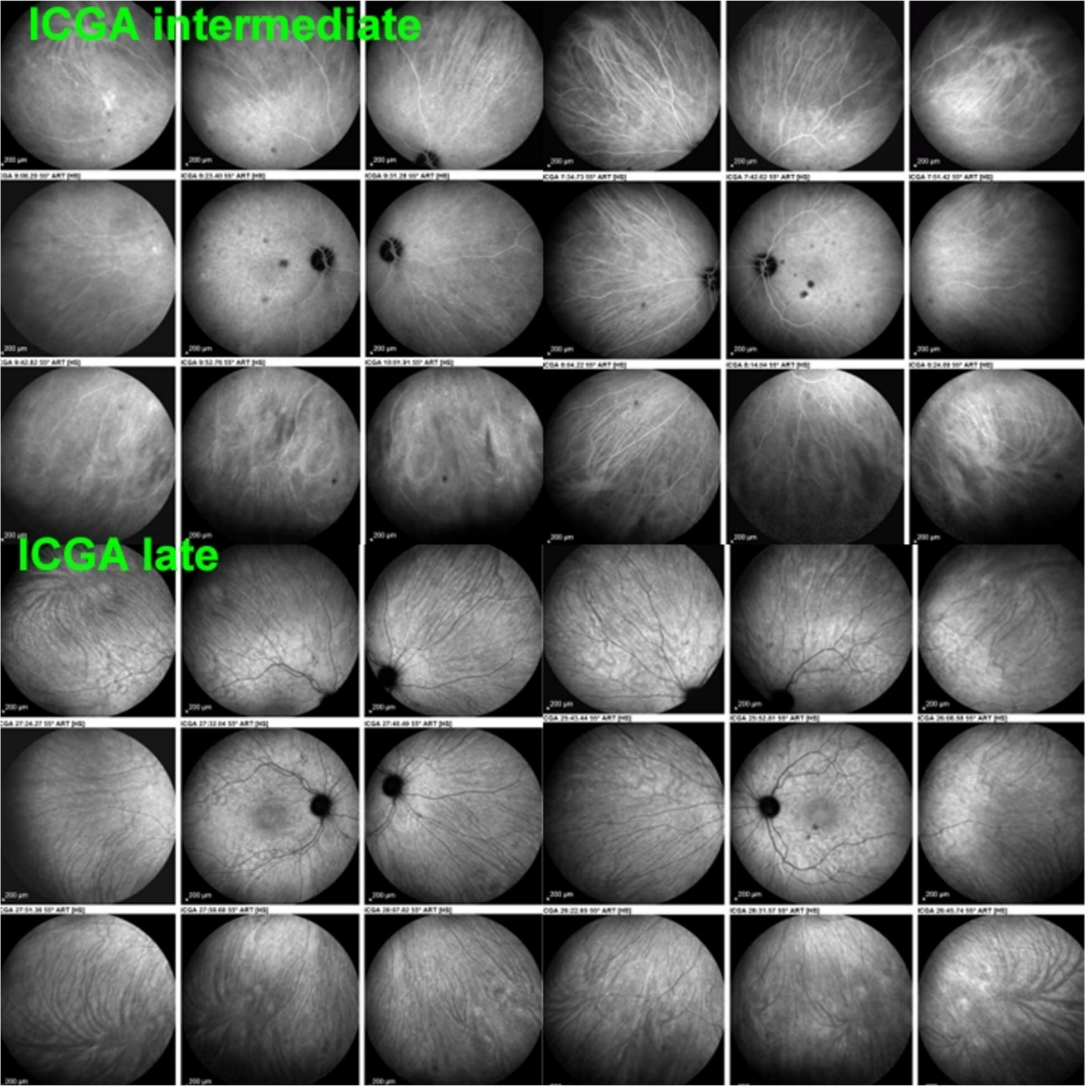
Ocular Sarcoidosis with minimal choroidal stromal vasculitis (score 0.5).This patient had preponderant retinal vasculitis (not shown) with minimal choroidal vasculitis. Several HDDs are seen in the posterior pole bilaterally in the intermediate angiographic phase (top 2 sets of nine frames). These HDDs disappear in the late angiographic phase (bottom 2 sets of 9 frames). Vessels are relatively well identified in the intermediate phase with choroidal vessels very discreetly fuzzy (2 top sets of 9 frames). In the late phase, choroidal vascular pattern is very distinctly identified in negative as the ICG dye is no more intravascularly and as there is no late hyperfluorescence that would obscure this pattern in case of more severe choroiditis (2 bottom sets of 9 frames)

In these entities, the disease does not start primarily from the choroidal structures such as in VKH or BRC, but lesions develop in the choroidal stroma secondarily as one of the random sites that can be involved by a systemic disease and is therefore called secondary stromal choroiditis (Fig. 34).

##### Stromal vasculitis in ocular sarcoidosis

**Fig. 35:**
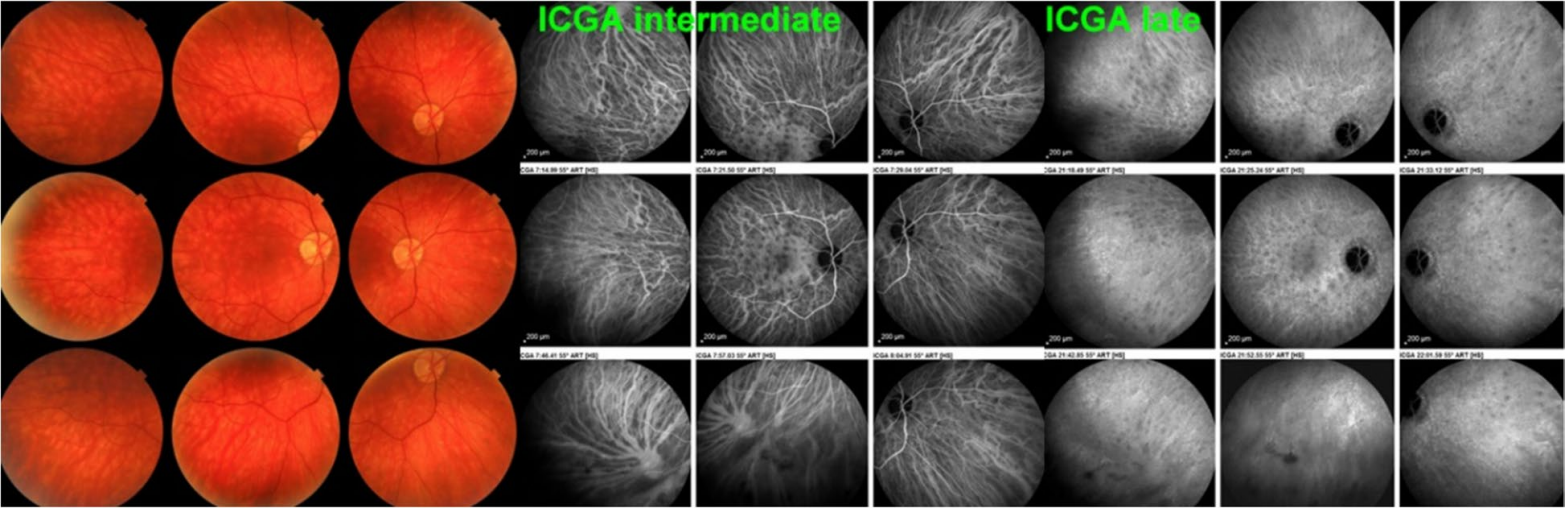
Bilateral Ocular Sarcoidosis with moderate choroidal stromal vasculitis (score 2, OD & OS, only OD is shown). This patient presented with bilateral faint round discoloured fundus lesions (left panfundus photography). BRC was suspected but HLA-A29 was absent and serum angiotensin converting enzyme (ACE) and lysozyme were elevated in addition to bilateral hilar lymphadenopathies (BHL). HDDs were clearly identified bilaterally on intermediate angiographic frames (middle set of 9 frames). However, vasculitis was minimal on intermediate frames as stromal vessels were well-identified with minimal fuzziness: Late phase angiographic frames (right set of 9 frames) however did not allow to identify stromal vessels because of late diffuse hyperfluorescence preventing identification of stromal vessels

**Fig. 36:**
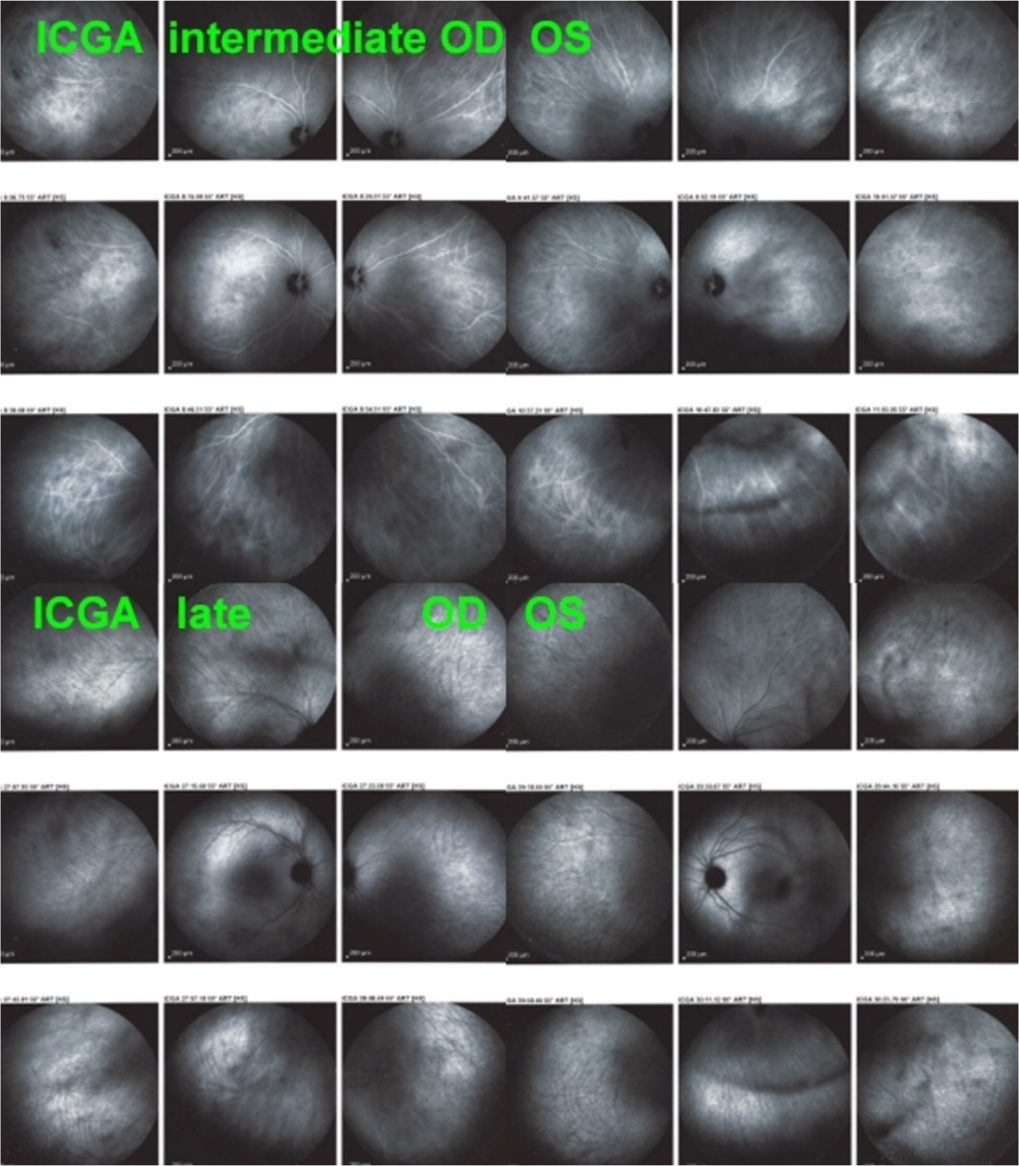
Bilateral Ocular Sarcoidosis with severe choroidal stromal vasculitis (score 6). This patient shows severe stromal choroidal vasculitis, as normal pattern of stromal vessels cannot be identified in the intermediate angiographic phase (top 2 sets of nine rames) nor in the late angiographic phase (bottom 2 sets of 9 frames) because of diffuse leakage from choroidal vessels. Note the irregular distribution and uneven size of HDDs, characteristic of sarcoidosis choroiditis

**Fig. 37:**
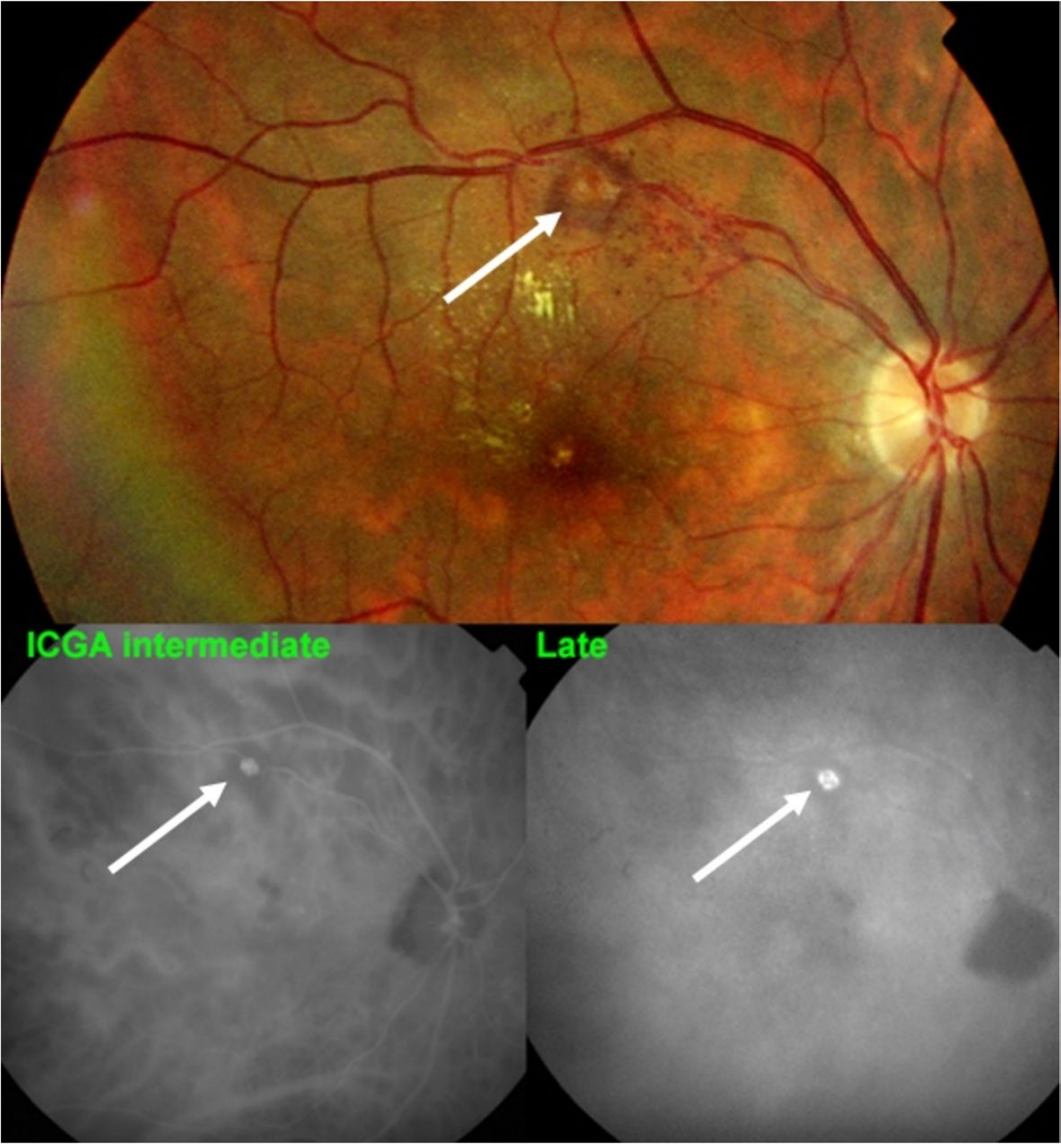
Ocular sarcoidosis with choroidal stromal vasculitis and macroaneurysm. This patient presented typical HDDs and substantial stromal vasculitis present in the intermediate and late angiographic phases in association with a retinal arterial macro-aneurysm OD (white arrows)

Ocular sarcoidosis is a chorioretinal inflammation involving both the retina and the choroid in different proportions. In up to 83% of cases the choroid is preponderantly involved [32]. As indicated, the choroidal involvement is considered a secondary stromal choroiditis as the systemic granulomatous disease process can involve the eye by chance and the choroidal location of lesions occurs randomly. Unlike in VKH, the repartition and size of hypofluorescent lesions in ICGA are uneven with often diverse involvement from one area of the fundus to another and from one eye to the other. To better define choroidal involvement, we analysed choroidal inflammation using the ASUWOG angiographic score [33]. Choroidal stromal vasculitis (fuzziness of vessels) is usually substantial, amounting to score of 3.09 ± 1.8 (with a maximal possible score of 6). In 3/23 patients the score was 0.5 or less and in 4/23 patients it was 5.5 or more. Examples of scores 0.5, 2 and 6 are given in Figs. 35, 36 and 37. Another characteristic vasculitic finding that can occur in sarcoidosis chorioretinitis is chorioretinal macro-aneurysm that is well identified by ICGA (Figs. 35, 36 and 37).

##### Stromal vasculitis in ocular tuberculosis (infectious)

**Fig. 38:**
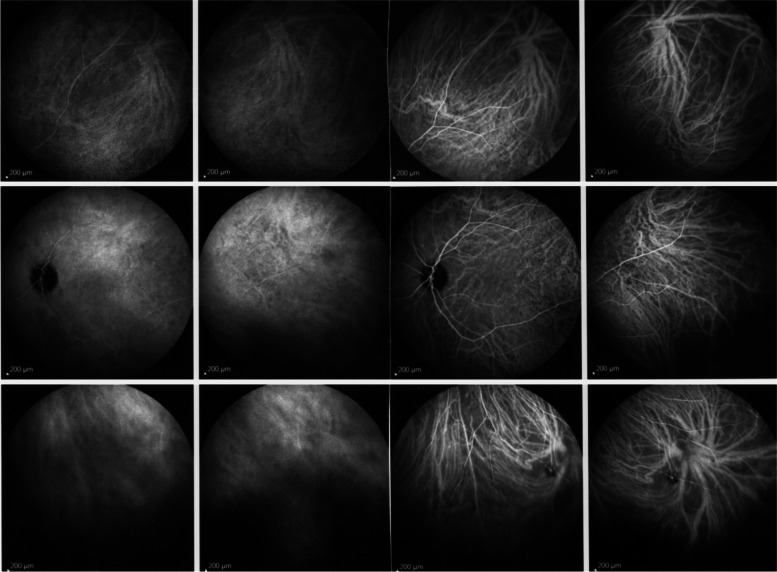
Tuberculous stromal vasculitis, intermediate angiographic phase (left eye). Patient shows substantial stromal vasculitis in the intermediate phase with unrecognizable stromal vessels and randomly distributed hypofluorescent areas of diverse sizes (left sextet of frames). After treatment the normal pattern of choroidal vessels can anew be identified and hypofluorescent areas have resolved (right sextet of frames)

Although tuberculous chorioretinitis is infectious, we will include, however, one example as the angiographic behaviour is comparable to sarcoidosis chorioretinitis because disease mechanisms are probably comparable. ICGA signs are most of the time undistinguishable in these two conditions [34] (Fig. 38).

##### Choroidal vasculitis in presence of choroidal tuberculoma

**Fig. 39:**
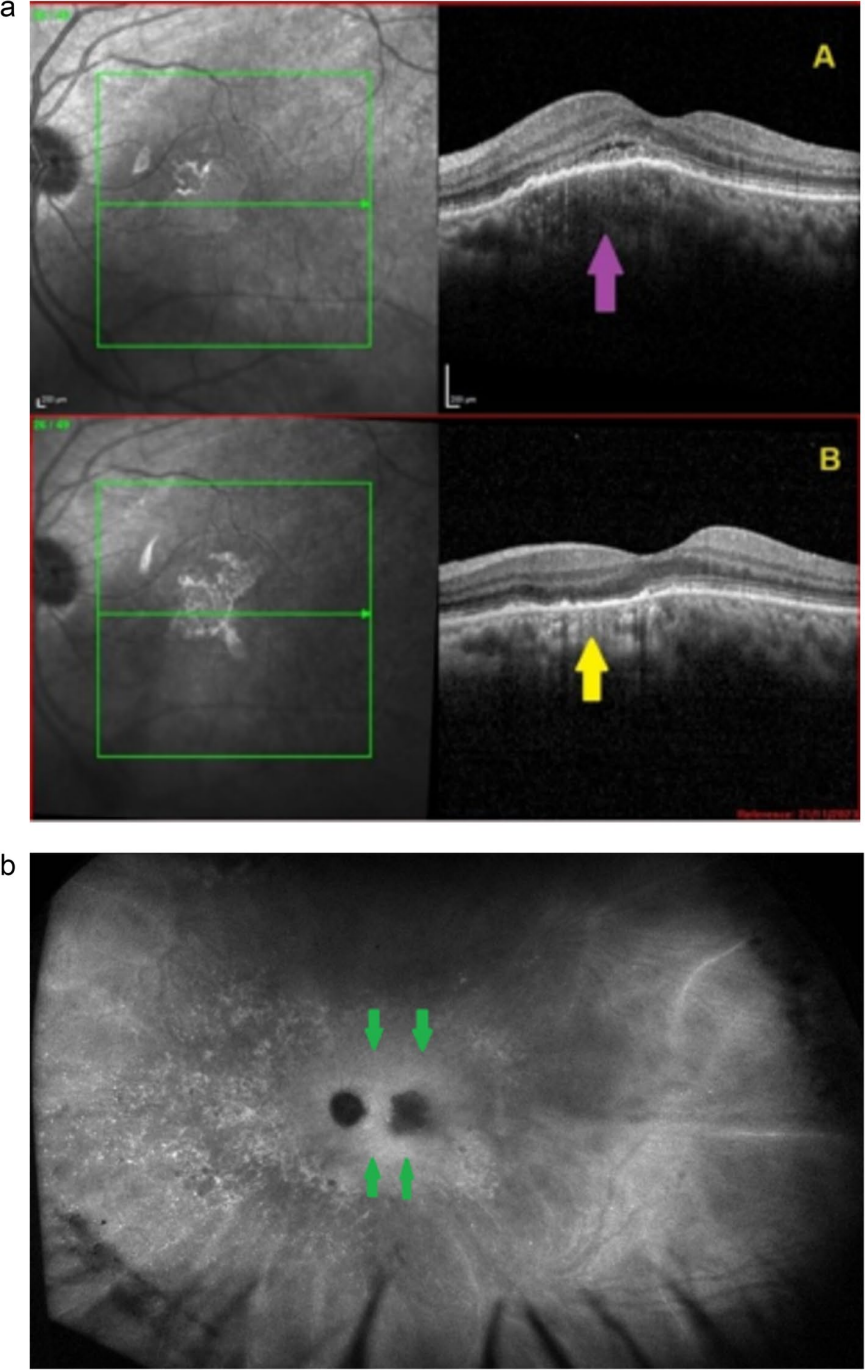
OCT of a tuberculoma. **A** OCT at presentation showing the tuberculoma (crimson arrow) as well as subretinal fluid due to damage to the outer retina because of mechanically induced choriocapillaris non perfusion. **B** OCT after systemic ATT and steroids were started. ICGA, intermediate phase, demonstrating a tuberculoma. The hypofluorescence is due to mechanically induced choriocapillaris non-perfusion. Note also perilesional hyperfluorescence due to vasculitis as well as diffuse fuzziness of vessels of the whole fundus

Another expression of choroidal vasculitis in ocular tuberculosis can be seen secondary to a choroidal granuloma or tuberculoma. A 59-year-old Indian lady consulted for vision loss in her left eye. OCT (Fig. 39A) showed the presence of a choroidal lesion compressing the choriocapillaris. ICGA showed choriocapillaris non-perfusion due to mechanical compression of the choroidal granuloma with perilesional hyperfluorescence due to choroidal vasculitis as well as the presence of diffuse fuzziness of choroidal vessels over the whole fundus (Fig. 39b). QuantiFERON was positive confirming the diagnosis of a tuberculoma. After standard ATT treatment and oral corticosteroids, the tuberculoma resolved (Fig. 39B).

#### Stromal choroidal vasculitis in systemic vasculitides

**Fig. 40:**
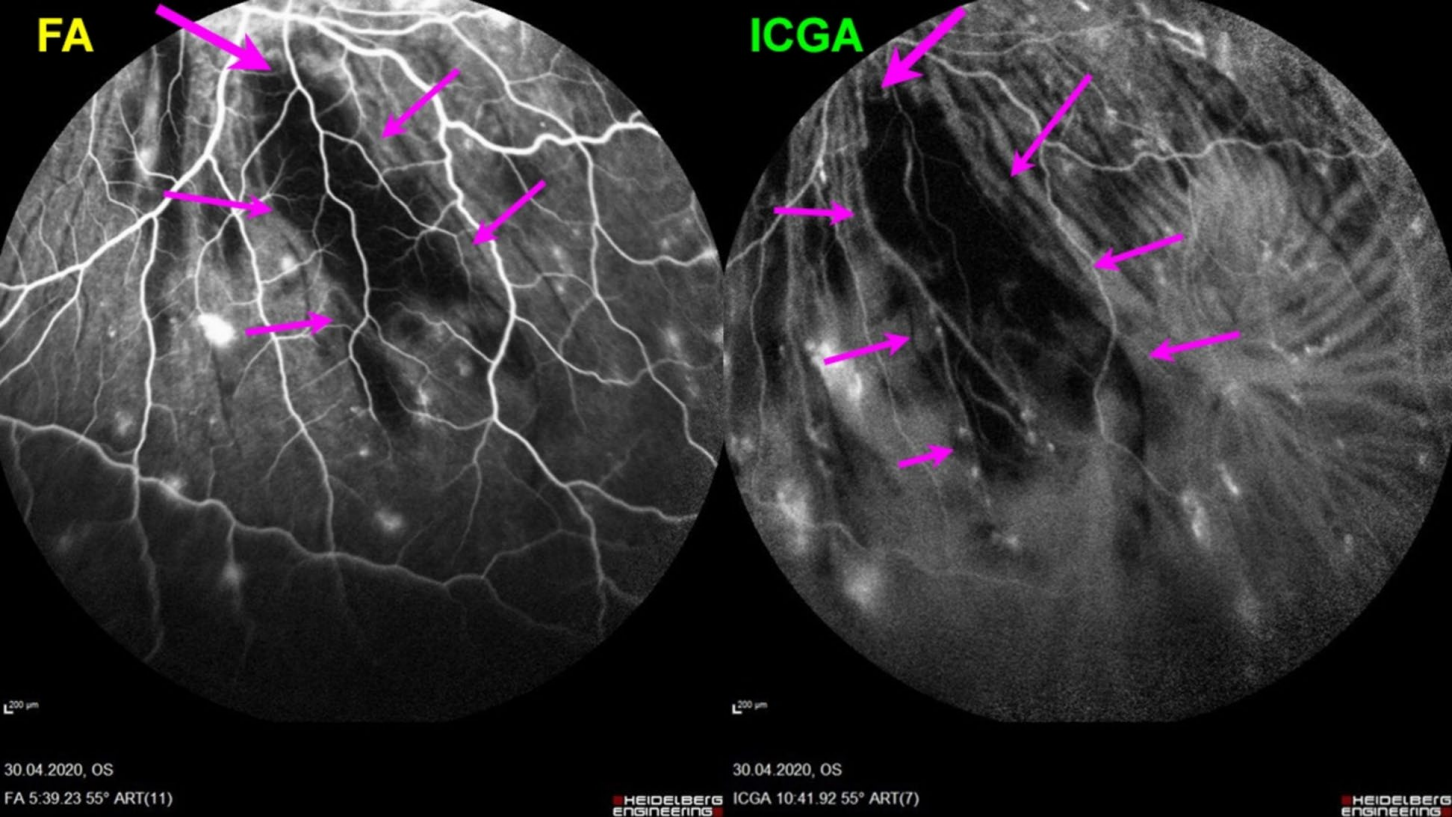
Systemic Lupus Erythematosus (SLE). Choroidal vasculitis in SLE is charcterised by extended drop-out of choroidal circulation including choriocapillaris due to vasculitic occlusion of a posterior ciliary artery. FA (left frame) shows drop out of choriocapillary circulation (crimson arrows) and ICGA shows non-perfusion of large choroidal vessels of a triangular area with apex towards the posterior pole typical of occlusion of short posterior ciliary arteries (Amalric’s sign). Images courtesy of Horst Helbig, University of Regensburg, Germany)

Systemic vasculitis and collagen diseases can be at the origin of choroidal vasculitis mostly in the form of choroidal ischemia due to occlusive involvement of choroidal vessels. Such occurrences have been described in systemic lupus erythematosus (SLE) and giant cell arteritis, and more rarely in polyarteritis nodosa, granulomatosis with polyangiitis (Wegener’s) [35], and Churg-Strauss syndrome [36]. Two examples on the choroidal vasculitis in systemic vasculitis will be given hereunder (Fig. 40).

##### Choroidal vasculitis in Systemic Lupus Erythematosus (SLE)

Choroidal vasculitis has been clearly described in an interesting article on ischemic choroidal diseases by Barth and Helbig [37]. In their case, FA showed choriocapillaris hypoperfusion in the posterior fundus and the peripheral sector corresponding to ischemic choroiditis. Occlusive choroidal vasculitis was clearly shown on ICGA in form of a triangular hypofluorescence with the apex towards the posterior pole corresponding to a non-perfused ischemic choroidal area, as first described by Amalric [38].

##### Choroidal vasculitis in Giant Cell Arteritis (GCA)

Giant-cell arteritis (GCA) or temporal arteritis or Horton’s arteritis is a generalized inflammatory disorder of unknown etiology involving large and medium-sized arteries preferably the cranial branches originating from the arc of the aorta with both systemic and ophthalmic manifestations. The disease occurs almost exclusively in people over age 50 and is more common among Caucasian populations with a greater incidence in women.

Diagnosis may be suggested by the existence of headache, jaw pain and signs of impaired general health (fever, asthenia, weight loss). Classical inflammatory indices such as C-reactive protein (CRP) and erythrocyte sedimentation rate (ESR) are considered key markers of the disease to date but the diagnostic gold standard is still the temporal artery biopsy showing arteritis. The incidence of reported visual complications varies widely from 20 to 70% [39]. About 20% of patients with ocular involvement are devoid of systemic symptoms (occult GCA) though in these cases the majority of patients have elevated ESR e CRP. GCA is characterized by granulomatous inflammatory infiltrates within the arterial walls with a predilection for involvement of the posterior ciliary arteries (PCA) causing vascular occlusion and tissue ischemia. The most common ocular signs are: acute ischemic optic neuropathy and choroidal ischemia; more rarely, vascular involvement comprises occlusion of the central retinal artery or of its branches and the presence of cotton-wool spots. FA but above all ICGA, reveal absence of choroidal filling of the area supplied by the occluded PCA. In this respect, the characteristic Amalric’s triangular sign indicates the infarction of a large area of choroidal tissue observed with the occlusion of the PCA [40]. Over time, this area becomes atrophic with some pigment clump accumulation in the fundus. Visual symptoms due to GCA represent an ophthalmic emergency and with an early diagnosis and an immediate and aggressive systemic corticosteroid therapy it is possible to avoid preventable visual loss in the fellow eye.

### Case report illustrating GCA

**Fig. 41:**
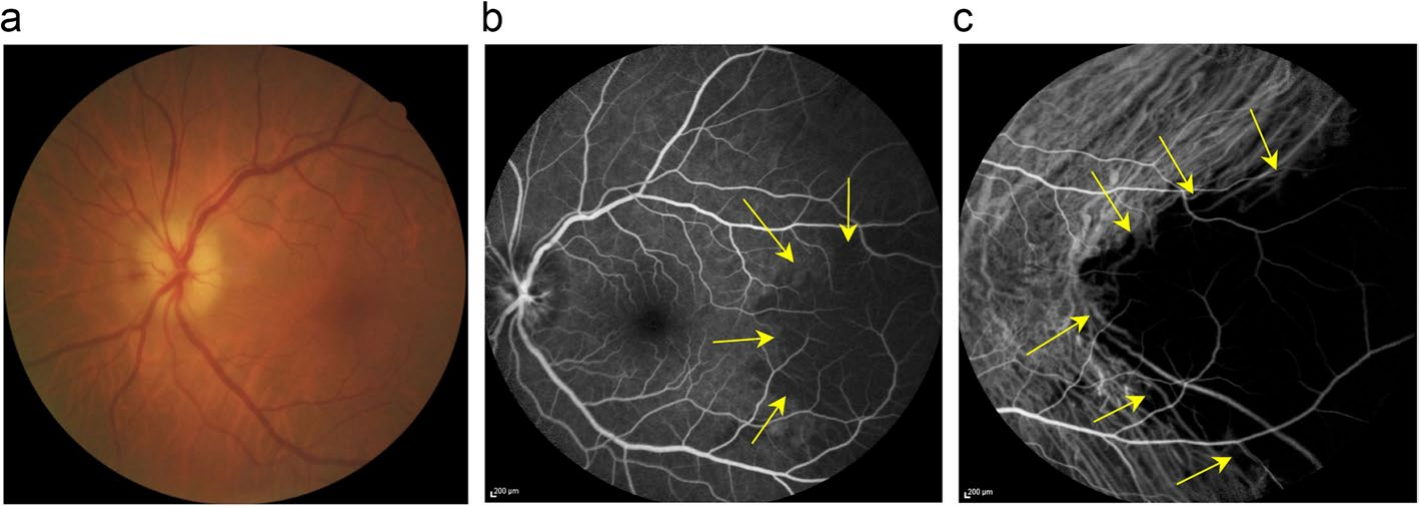
**a **Fundus picture shows a chalky white optic nerve head typical of arteritic anterior ischemic optic neuropathy. **b** Early fluorescein angiography (FA early) shows a faint hypofluorescent triangle in the temporal periphery (yellow arrows) due to choroidal hypo-perfusion. **c** Indocyanine green Angiography (ICGA) clearly shows the triangular sign of Amalric due to choroidal and choriocapillaris non-perfusion of the posterior short ciliary artery (yellow arrowheads)

An 80-year-old woman presented with sudden onset of diminution of vision in her left eye for one day, preceded by an episode of amaurosis fugax. Laboratory investigations showed an ESR of 89 mm/h and a CRP of 2.5 mg/dL. A biopsy of the left superficial temporal artery was performed and the diagnosis of GCA was confirmed. On fundoscopy, a chalky white optic disc edema indicated anterior ischemic optic neuropathy (AION) (Fig. 41a) and a triangular retinal whitening in the midperipheral temporal fundus indicating a previous posterior choroidal artery occlusion. FA showed delayed choroidal filling in the early phase (Fig. 41b) followed by late hyperfluorescence due to rupture of the outer retinal barrier. ICGA indicated a large triangular area of choroidal nonperfusion with triangle vertex toward the posterior pole, consistent with a diagnosis of PCA occlusion (Fig. 41c).

The patient was promptly treated with intravenous 1 gr of Prednisone followed by 1 mg/Kg/day of oral Prednisone on a careful tapering schedule.

### Choroidal stromal vasculitis related to scleritis

Vermeirsch et al. recently published a review about choroidal involvement in non-infectious scleritis and choroidal vasculitis was among the features [41]. Choroidal vasculitis was also proved histopathologically. Calthorpe et al. showed that histopathological examination of scleral biopsies of enucleated eyes of patients with posterior scleritis revealed choroidal vasculitis in 57% of their cases [42]. 

## Discussion

Choroidal vasculitis is a sign of inflammation of the choroid frequently seen in posterior uveitis involving the choroid. The pathophysiology and angiographic signs of choroidal vasculitis should not be interpreted in the same way as fluorescein angiographic interpretation of retinal vasculitis. This is because choroidal vessels, including large stromal vessels and choriocapillaris, have different characteristics. Choriocapillaris is a network of fenestrated capillaries with low flow especially in small end-choriocapillaris vessels whereas stromal vessels are impermeable due to tight junctions. For that reason, choroidal vasculitis is divided into stromal choroidal vasculitis and choriocapillary vasculitis / choriocapillaritis with different signs, different prognosis and different management between the pathologies in each entity. It is obvious that the smaller the vessel affected the lesser the symptoms and signs. In order to identify which type of vessel is affected, an ICGA is necessary.

ICGA made it possible not only to detect choroidal vasculitis but allowed to adequately classify non-infectious choroiditis. Precise semiology of ICGA signs of non-infectious choroiditis could be established that are reported in this article. These angiographic signs are biomarkers of choroidal inflammation and are essential to monitor disease evolution and impact of therapy. Since the availability of ICGA in the mid1990ties, new imaging modalities such as EDI-OCT, OCT-A and FAF have contributed additional information on choroidal inflammation with ICGA remaining the principal modality giving global information on vasculitis and inflammatory involvement of the choroid.

Choroidal involvement and vasculitis in posterior uveitis have often been neglected by clinicians, especially when ICGA is not performed. The hypofluorescent areas seen on ICGA, are characteristic of choriocapillaris involvement produced by inflammatory choriocapillaris non-perfusion producing a patchy or geographic pattern in case of choriocapillaritis. In contrast, HDDs in stromal choroiditis are explained by space-occupying lesions that do not allow the diffusion of the ICG dye and take the aspect of regular roundish evenly distributed dots in primary stromal choroiditis entities such as VKH disease and BRC. The ICGA findings are not limited to these hypofluorescent signs usually reported but interpretation has to also take into account the signs of stromal vasculitis that translate into vessel hyperfluorescence described as fuzzy vessels at the origin of late diffuse hyperfluorescence, two additional ICGA signs described early on by our group [6]. These signs alongside the HDDs, help determine the severity of disease and monitor the effect of therapeutic decisions in stromal choroiditis. As shown in our cases, the presence on ICGA of choroidal fuzziness, late hyperfluorescence and optic disc hyperfluorescence is more frequently seen in VKH disease than in BRC. ICGA is thus able to identify the severity of choroidal inflammation and determine therapeutic intervention.

On the other hand, choriocapillaritis/ choriocapillary vasculitis is affecting much smaller vessels than stromal choroiditis and causing their inflammatory closure. In this respect ICGA also represents the gold standard to identify the type of vessel affected. Larger choriocapillaris vessels are affected in SC and MFC and aggressive treatment is recommended while smaller vessels are affected in APMPPE and the smallest end-capillaries in MEWDS. These pathophysiological differences explain why no treatment is needed in MEWDS and in some APMPPE cases.

Investigation of posterior uveitis was only partial until access to the choroidal circulation was possible using ICGA and, hence, choroidal vasculitis could be determined. FA allowed to precisely determine retinal vasculitis and represented a substantial progress since its first description by Novotny and Alvis in the early 1960ties [43]. Being limited to the analysis of the retinal circulation, FA was improper to investigate posterior inflammatory diseases developing in the choroid. Only a glimpse of choriocapillaris circulation was obtained by FA in the first 60 s of the angiographic sequence. Therefore, in the absence of ICGA, it was difficult to characterise the mechanisms of several diseases that were being described from the 1960ties to 1990ties, including APMPPE, MEWDS, BRC and others. The miscomprehension of these entities led to the emergence of the useless terminology of white dot syndromes (WDS) grouping diseases merely on the basis of fundus appearance, telling absolutely nothing on the location and clinicopathology of these diseases. A more appropriate classification of choroidal vasculitis based on ICGA is now available for non-infectious choroiditis diseases. Furthermore, inflammation in this compartment can now be reliably followed and treated.

## Data Availability

No datasets were generated or analysed during the current study.

## Abbreviations

ICGA: indocyanine green angiography
MFC: multifocal choroiditis
APMPPE: acute posterior multifocal pigment epitheliopathy
MEWDS: Multiple evanescent white dots syndrome
RPE: retinal pigment epithelium
HDDs: hypofluorescent dark dots
OCT-A: Optical coherence tomography-angiography
SC: serpiginous choroiditis
VKH: vogt-koyanagi-harada
BRC: Birdshot retino-choroiditis
SD-OCT: Spectral Domain Optical Coherence Tomography
EDI -OCT: Enhanced Depth Imaging OCT
FAF: fundus autofluorescence
SO: Sympathetic Ophthalmia
ASPPC: Acute Syphilitic Posterior Placoid Chorioretinitis
ATT: anti-tuberculous treatment
SLE: systemic lupus erythematosus
GCA: Giant-cell arteritis
CRP: C reactive protein
ESR: erythrocyte sedimentation rate
PCA: posterior ciliary arteries
AION: anterior ischemic optic neuropathy
